# Differential aortic aneurysm formation provoked by chemogenetic oxidative stress

**DOI:** 10.1172/JCI188743

**Published:** 2025-03-18

**Authors:** Apabrita Ayan Das, Markus Waldeck-Weiermair, Shambhu Yadav, Fotios Spyropoulos, Arvind Pandey, Tanoy Dutta, Taylor A. Covington, Thomas Michel

**Affiliations:** 1 Cardiovascular Medicine Division, Brigham and Women’s Hospital, Boston, Massachusetts, USA.; 2 Harvard Medical School, Harvard University, Boston, Massachusetts, USA.; 3 Molecular Biology and Biochemistry, Gottfried Schatz Research Center, Medical University of Graz, Graz, Austria.; 4 Department of Pediatrics, Brigham and Women’s Hospital, Boston, Massachusetts, USA.

**Keywords:** Cardiology, Vascular biology, Cardiovascular disease

## Abstract

Aortic aneurysms are potentially fatal focal enlargements of the aortic lumen; the disease burden is increasing as the human population ages. Pathological oxidative stress is implicated in the development of aortic aneurysms. We pursued a chemogenetic approach to create an animal model of aortic aneurysm formation using a transgenic mouse line, DAAO-TG^Tie2^, that expresses yeast d-amino acid oxidase (DAAO) under control of the endothelial Tie2 promoter. In DAAO-TG^Tie2^ mice, DAAO generated the ROS hydrogen peroxide (H_2_O_2_) in endothelial cells only when provided with d-amino acids. When DAAO-TG^Tie2^ mice were chronically fed d-alanine, the animals became hypertensive and developed abdominal, but not thoracic, aortic aneurysms. Generation of H_2_O_2_ in the endothelium led to oxidative stress throughout the vascular wall. Proteomics analyses indicated that the oxidant-modulated protein kinase JNK1 was dephosphorylated by the phosphoprotein phosphatase DUSP3 (dual specificity phosphatase 3) in abdominal, but not thoracic, aorta, causing activation of Kruppel-like Factor 4 (KLF4)-dependent transcriptional pathways that triggered phenotypic switching and aneurysm formation. Pharmacological DUSP3 inhibition completely blocked the aneurysm formation caused by chemogenetic oxidative stress. These studies establish that regional differences in oxidant-modulated signaling pathways lead to differential disease progression in discrete vascular beds and identify DUSP3 as a potential pharmacological target for the treatment of aortic aneurysms.

## Abstract 2

<span>Aortic aneurysms are potentially fatal enlargements in the lumen of the aorta. </span> These studies have developed a new animal model that was exploited to understand the mechanisms whereby aneurysms form, and to identify potential new targets for drug treatment.

## Graphical abstract

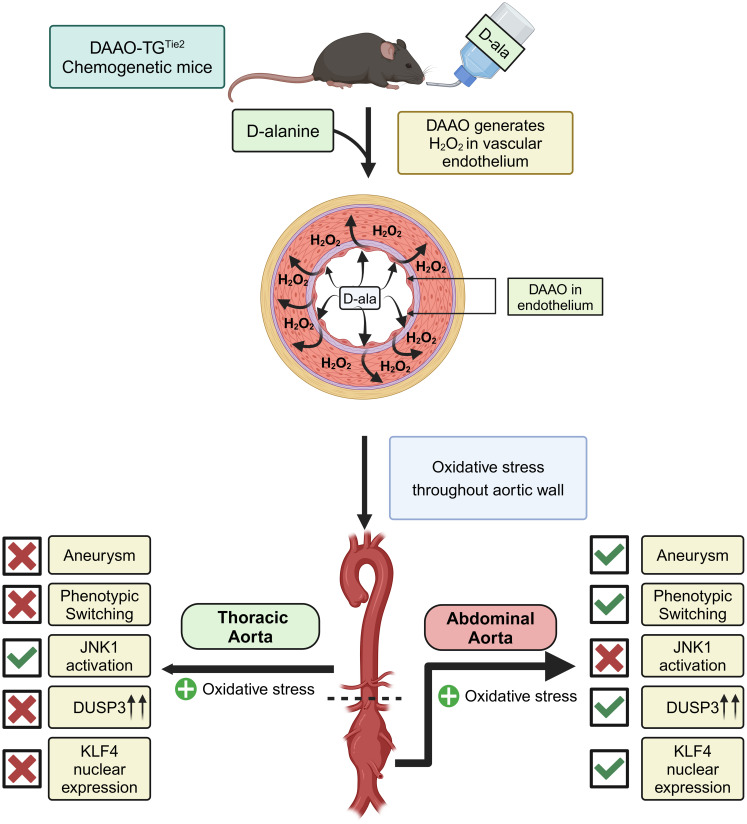

## Introduction

Elevated levels of ROS in the vasculature have long been linked to hypertension and aortic aneurysm formation associated with pathological oxidative stress (1–7). It is unclear whether oxidative stress plays a causal role in the development of hypertension or aortic aneurysms, or whether oxidative stress is instead merely associated with these complex disease states. Many of the current animal models of aortic aneurysm formation and hypertension are hampered by methodological complexities, and yet other models may not replicate the molecular mechanisms implicated in disease progression in humans (8, 9). Here we used a chemogenetic approach (10, 11) to develop an animal model that leverages a key feature shared by many of the disease states that lead to hypertension and aneurysm formation: oxidative stress in the vasculature.

Chemogenetic approaches utilize recombinant proteins that are selectively activated by unique ligands or substrates to elicit specific responses in target cells (12). Intracellular redox balance can be dynamically regulated using chemogenetic approaches exploiting a recombinant yeast d-amino acid oxidase (DAAO) that can be activated to generate hydrogen peroxide (H_2_O_2_) (12). Yeast DAAO is a stereospecific enzyme that generates H_2_O_2_ as part of its catalytic scheme involving the oxidation of d-amino acids to their corresponding α-keto acids; here, we use d-alanine as the DAAO substrate (10, 11, 12). In addition to producing H_2_O_2_, DAAO generates equimolar ammonia and pyruvate, but the intracellular concentrations of these other DAAO products are much higher than H_2_O_2_ levels; thus, cellular levels of ammonia (13) and pyruvate are not substantively affected by DAAO catalysis (14). Most mammalian tissues contain l- but not d-amino acids, so the recombinant DAAO is inactive until d-alanine is provided to cells expressing DAAO. We previously generated and characterized transgenic mouse lines that express DAAO under control of tissue-specific promoters such that the addition of d-amino acids to the animals’ drinking water activates DAAO in target tissues, thereby increasing cellular H_2_O_2_ and causing oxidative stress (10, 11). Studies of these “transgenic/chemogenetic” mouse lines have facilitated the analysis of pathways whereby oxidative stress influences disease pathogenesis, and led to the development of novel animal models of neurodegeneration and heart failure caused by tissue-specific chemogenetic oxidative stress in neurons (11) and cardiac myocytes (10), respectively. Here we focus on studies using the DAAO-TG^Tie2^ mouse, in which DAAO is expressed under control of the endothelial cell–specific Tie2 promoter, aiming to define the effects of endothelial cell–specific oxidative stress on the development of hypertension and aortic aneurysms.

Aortic aneurysms are focal enlargements in the lumen of the aorta and represent a major cause of morbidity and mortality in patients (15–17). There are marked differences in the natural history of and risk factors for aortic aneurysms arising in the thoracic aorta (starting at the aortic valve and ending at the diaphragm) versus the abdominal aorta (from the diaphragm to the aortic bifurcation). These two aortic regions have distinct embryological origins and are subject to different hemodynamic forces (18), and different clinical risk factors affect the development of abdominal and thoracic aneurysms (17, 19, 20). Pathological oxidative stress has been implicated in the development of both thoracic and abdominal aorta aneurysms, yet the molecular mechanisms underlying the regional differences in aneurysm formation are incompletely understood. Our current studies of the DAAO-TG^Tie2^ mouse establish that vascular oxidative stress is necessary but not sufficient for aneurysm formation and identify the molecular pathways that underlie the differential responses of abdominal versus thoracic aorta to endothelial oxidants.

## Results

### The chemogenetic/transgenic DAAO-TG^Tie2^ mouse line.

We generated a chemogenetic transgenic mouse line that expresses DAAO in vascular endothelial cells to study the effects of chronic oxidative stress on the vasculature. DAAO-TG^LoxP^ mice (11) were crossed with a mouse line expressing Cre recombinase under control of the endothelium-specific Tie2 promoter (The Jackson Laboratory). Founder lines were isolated to create the DAAO-TG^Tie2^ mouse line (Supplemental Figure 1, A and B; supplemental material available online with this article; https://doi.org/10.1172/JCI188743DS1). The Tie2 promoter has been characterized: this promoter is highly active in vascular endothelial cells, but there is also some Tie2 promoter–driven transgene expression in hematopoietic cells (21, 22).

### Effects of chronic oxidative stress on d-alanine–fed DAAO-TG^Tie2^ mice.

**Figure 1:**
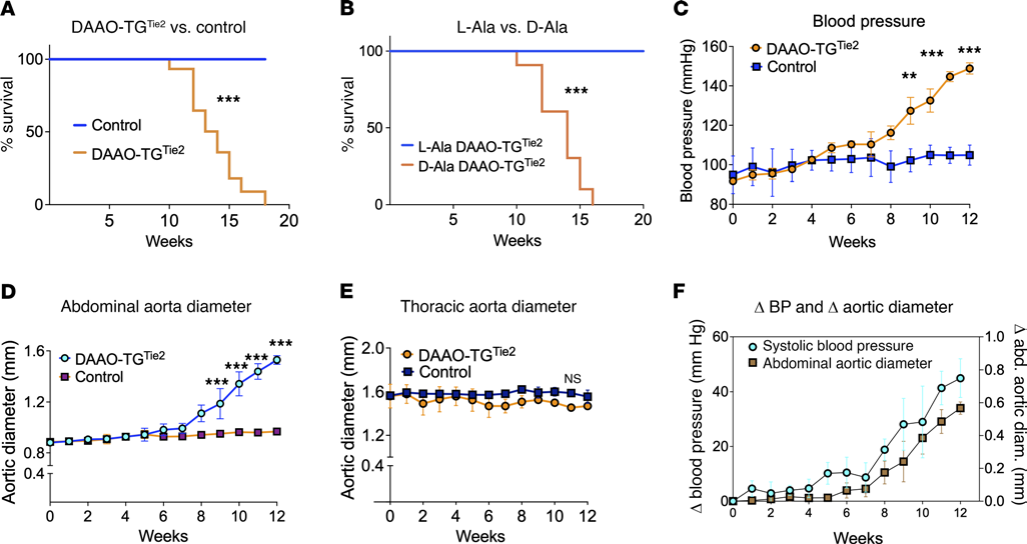
Effects of chemogenetic oxidative stress on mortality and vascular pathology in DAAO-TG^Tie2^ transgenic mice. (**A** and **B**) Kaplan-Meier survival curves for DAAO-TG^Tie2^ and control mice exposed to chemogenetic oxidative stress. (**A**) Survival curve for d-alanine–fed DAAO-TG^Tie2^ transgenic mice (blue line), with their wild-type d-alanine–fed littermates serving as negative (genetic) controls (orange line). (**B**) Treatment control in which DAAO-TG^Tie2^ transgenic mice were fed either d-alanine (blue line) or l-alanine (orange line). Chronic d-alanine–fed DAAO-TG^Tie2^ transgenic mice show drastically reduced survival compared either with their d-alanine–fed wild-type control littermates (*P* < 0.001 by ANOVA) or with l-alanine–fed transgenic littermates (*P* < 0.001). (**C**) Results of systolic blood pressure measurements in DAAO-TG^Tie2^ transgenic (blue line) and their wild-type littermates (orange line) following the initiation of d-alanine feeding; there is no change in blood pressure until 8 weeks of age, at which point blood pressure increases significantly (**P < 0.01, ***P < 0.001, ANOVA). (**D** and **E**) Measurements of aortic diameter (determined by sonography) in d-alanine–fed DAAO-TG^Tie2^ transgenic mice (blue line) and their wild-type littermates (orange line) are shown for the abdominal aorta (**D**) (***P < 0.001) and the thoracic aorta (**E**). In the DAAO-TG^Tie2^ transgenic mice, abdominal (*P* < 0.001) but not thoracic aortae increase in size following chronic d-alanine feeding. (**F**) Time course plotting the change in systolic blood pressure and the change in abdominal aorta dimensions following the initiation of d-alanine feeding in DAAO-TG^Tie2^ transgenic animals compared with wild-type littermate controls; changes in blood pressure and abdominal aortic dimension followed similar time courses. All measurements were made by observers blinded to genotype and treatment. All mice were on a similar C57BL/6 genetic background. Data are presented as means ± SEM of at least 3 independent experiments.

In our previous studies of transgenic mice expressing DAAO in cardiac myocytes (10) or sensory neurons (11), we found that d-alanine treatment produced a striking disease phenotype within a few days or weeks of treatment. As we had done previously, we provided DAAO-TG^Tie2^ transgenic and control mice with 0.75 M d-alanine in their drinking water, and then monitored the animals carefully, anticipating the development of a vascular phenotype (Supplemental Figure 1C). All measurements were determined by observers blinded to treatment and genotype. We used 2 different controls: We studied a *genetic control*, comparing transgene-positive DAAO-TG^Tie2^ mice with their transgene-negative Cre^+^ (Cre^+^TG^–^) littermates, both of which were fed d-alanine (Figure 1A). We also performed a *treatment control*, in which DAAO-TG^Tie2^ mice were treated with either d-alanine or l-alanine (Figure 1B). For the first 2 months of treatment, the animals appeared to be healthy, and weekly blood pressure measurements (tail cuff) and aortic sonography showed no change. However, following 2 months of treatment, we observed marked increases in systolic blood pressure (Figure 1C) and in the diameter of the abdominal aorta (Figure 1D) in the DAAO-TG^Tie2^ mice that had been provided with d-alanine, with no change in the diameter of their thoracic aorta (Figure 1E). In all control animals, the systolic blood pressure and aortic dimensions remained unchanged. After 3 months, we observed a sharp decline in the survival of DAAO-TG^Tie2^ mice subjected to d-alanine treatment; control mice remained viable and healthy (Figure 1). The development of aneurysmal dilatation of the abdominal aorta and the onset of hypertension showed a similar time course (Figure 1F). The d-alanine–fed DAAO-TG^Tie2^ mice appeared healthy, and then died suddenly after 3 months; necropsies performed on d-alanine–fed DAAO-TG^Tie2^ mice revealed that in each case (*n* = 16) the abdominal cavity was filled with blood, suggestive of a vascular catastrophe.

### Comparison of transgene expression in abdominal and thoracic aorta.

**Figure 2:**
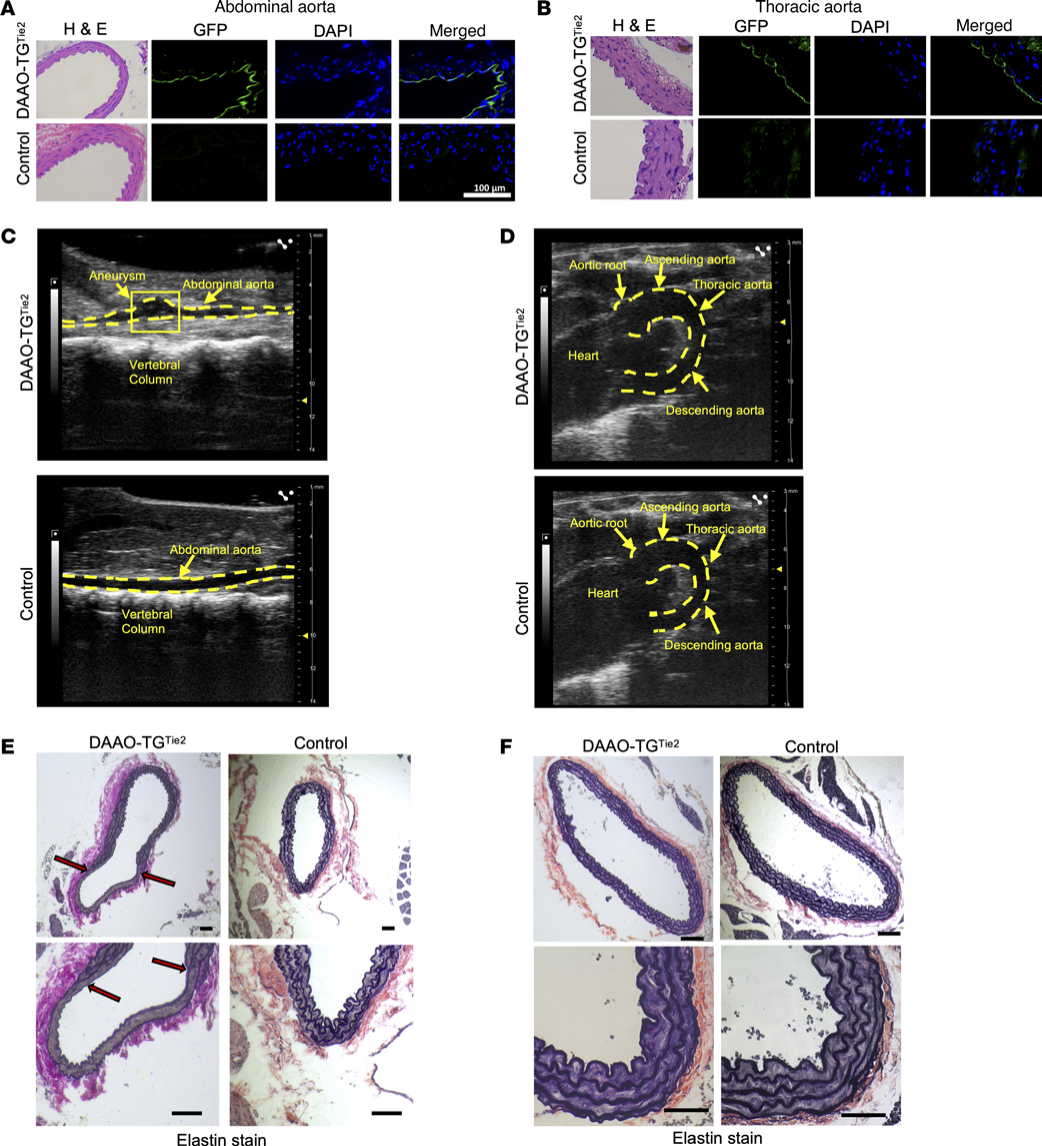
Imaging of the abdominal and thoracic aorta following chemogenetic oxidative stress. The figure shows representative images of abdominal (**A**, **C**, and **E**) and thoracic (**B**, **D**, and **F**) aortae from DAAO-TG^Tie2^ transgenic and control mice. (**A** and **B**) Aortae were isolated from untreated DAAO-TG^Tie2^ transgenic mice or control littermates, and transverse sections were stained with hematoxylin and eosin (H&E); with antibodies against GFP (which detects the YFP that is part of the transgene); or with DAPI to detect cell nuclei. The final panel in each row shows the merged image of GFP and DAPI staining. Expression of the transgene is seen in both abdominal and thoracic aorta in the transgenic mice. Scale bar: 100 μm. (**C** and **D**) Representative aortic sonograms are shown for infrarenal abdominal (**C**) and thoracic (**D**) aorta from d-alanine–fed DAAO-TG^Tie2^ transgenic and control mice. The dashed lines in each image show the border of the aortic lumen, revealing an aneurysm in abdominal (infrarenal) but not thoracic aorta. The aortic root and the ascending and descending part of the thoracic aorta are denoted by arrows. (**E** and **F**) Images of abdominal (**E**) and thoracic (**F**) aortae from d-alanine–treated DAAO-TG^Tie2^ or control mice. Fixed aortic sections were stained with Van Gieson’s elastin stain. The top and bottom panels show lower and higher magnifications, and the scale bars designate 40 μm or 10 μm, respectively. An aortic aneurysm can be seen in the abdominal (infrarenal) but not thoracic aorta (descending) sections. Red arrows indicate start and end of aneurysmal bulge in the corresponding lower- and higher-resolution images. The images in this figure are representative of at least 3 independent experiments.

A trivial explanation for the difference in aneurysm formation between thoracic and abdominal aorta is that there is differential expression of the DAAO-TG^Tie2^ transgene between these two vascular beds. We stained vascular tissues from transgenic and control mice with antibodies against GFP, which detects the YFP component of the HyPer biosensor in the DAAO-TG^Tie2^ transgenic fusion construct. We found that the transgene is expressed at similar levels in abdominal (Figure 2A) and thoracic aorta (Figure 2B), despite the marked difference in the abdominal aortic dimensions over time following d-alanine treatment. Histopathological staining documented the presence of aortic aneurysms only in the infrarenal portion of the abdominal aorta but not in the thoracic aorta (Figure 2, C and D, and Supplemental Figure 2, A and B). Thinning and bulging of the abdominal aortic wall were revealed by elastin staining (Figure 2, E and F, and Supplemental Figure 2, A and B) with an increased abdominal lumen circumference in treated DAAO-TG^Tie2^ compared with treated controls (Supplemental Figure 2C). Aortic sonography identified aneurysm formation (>50% increase in aortic diameter) only in the infrarenal abdominal aorta (Figure 2C) in d-alanine–fed DAAO-TG^Tie2^ mice; male and female mice were affected equally (Supplemental Table 1). The thoracic aorta showed no signs of aneurysm formation in d-alanine–fed DAAO-TG^Tie2^ mice (Figure 2D, Supplemental Figure 2D, and Supplemental Videos 1 and 2). Levels of H_2_O_2_ formation in aortic tissues were analyzed using the Amplex Red assay (23) either in d-alanine–fed or in untreated DAAO-TG^Tie2^ mice (Supplemental Figure 3A). d-Alanine feeding caused an increase in the Amplex Red signal in both thoracic and abdominal aorta in d-alanine–fed DAAO-TG^Tie2^ mice compared with untreated mice, but there was no difference in the Amplex Red signal comparing thoracic versus abdominal aorta. Levels of transgene protein expression were analyzed in immunoblots, and showed no differences between thoracic and abdominal aortae (Supplemental Figure 3B). Fibrosis, elastin degradation, and extracellular matrix degradation were documented in the abdominal but not thoracic aorta using histochemical stains (Supplemental Figure 4). We also performed immunostaining in abdominal aortic tissues from d-alanine–treated DAAO-TG^Tie2^ mice using antibodies against a range of “inflammatory” cells, including CD3 (to detect T cells); CD45 (B cells); CD11c (dendritic cells); CD68 (monocytes); F4/80 (macrophages); and Ly6C (neutrophils). None of the antibodies identified any increase in these inflammatory cells in the aorta analyzed after 3 months of d-alanine feeding (Supplemental Figure 5).

### Bone marrow transplantation experiments to differentiate hematopoietic versus endothelial cell expression of the Tie2-driven transgene in aortic aneurysm formation.

The “endothelial cell–specific” Tie2 promoter also drives gene expression in hematopoietic cells (21, 22). We irradiated DAAO-TG^Tie2^ and Cre^+^TG^–^ control mice to ablate hematopoietic cells, and performed bone marrow transplants from non-irradiated donor mice, and then treated the transplant recipients with d-alanine (Supplemental Figure 6A). Bone marrow transplanted from untreated DAAO-TG^Tie2^ mice into irradiated control littermates did not result in any vascular phenotype in wild-type transplant recipients treated with d-alanine (Supplemental Figure 6, B and C). By contrast, ablation of bone marrow in DAAO-TG^Tie2^ mice did not prevent the subsequent development of abdominal aortic aneurysms in response to d-alanine feeding after transplantation of bone marrow from unirradiated control mice (Supplemental Figure 6D). We conclude that expression of the Tie2-driven DAAO transgene in vascular endothelial cells, and not in hematopoietic cells, is responsible for the vascular phenotype.

### Oxidative stress in the abdominal and thoracic aorta.

**Figure 3:**
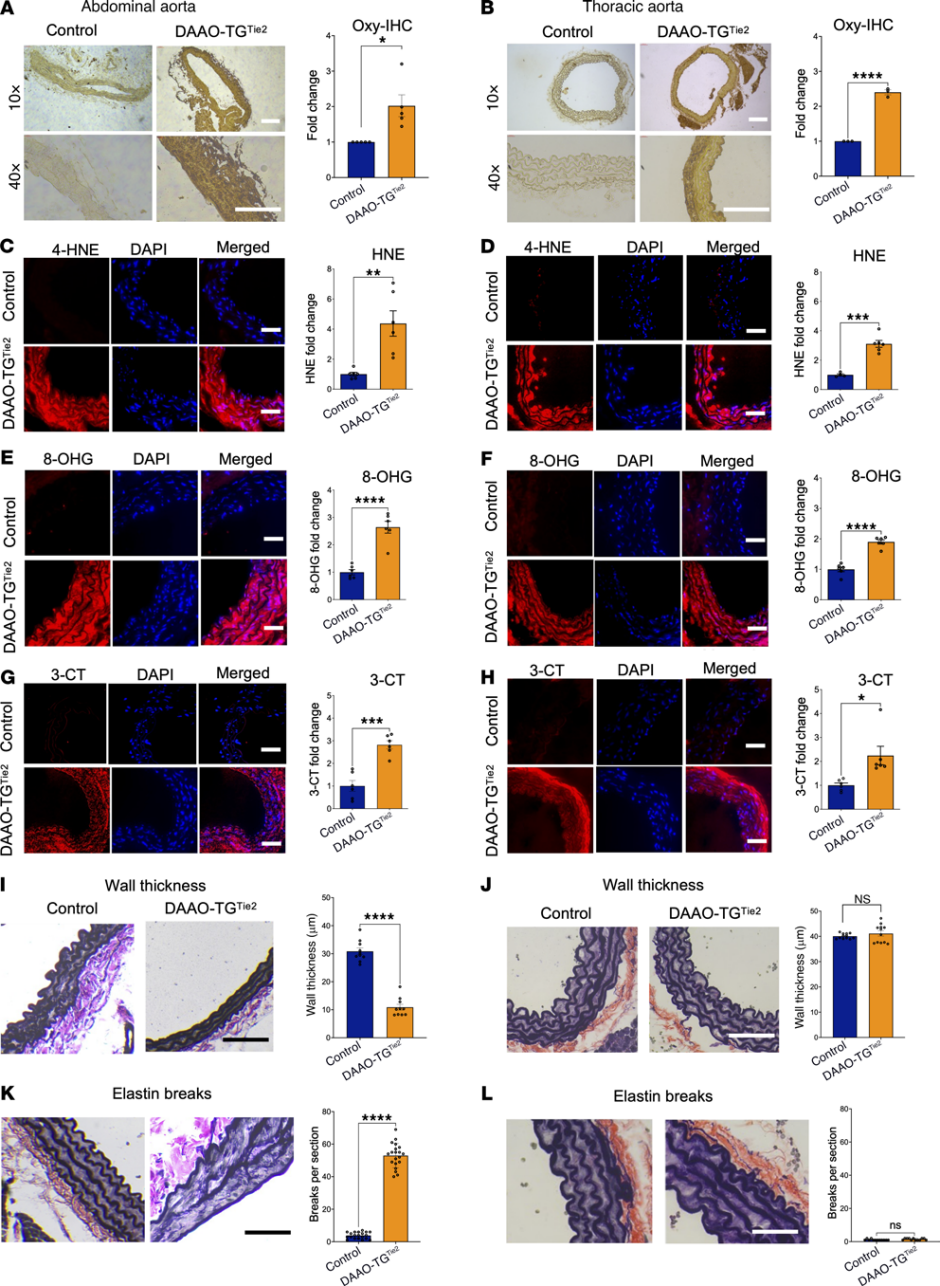
Analyses of aortic wall oxidative stress and vessel integrity in abdominal and thoracic aortae isolated from d-alanine–fed DAAO-TG^Tie2^ transgenic and control mice. DAAO-TG^Tie2^ transgenic and control mice were treated with d-alanine for 2 months, and fixed aortic preparations were isolated and analyzed to quantitate markers of oxidative stress and vessel integrity. Throughout this figure, images from abdominal aorta are shown in the left panels (**A**, **C**, **E**, **G**, **I**, and **K**) and images from thoracic aorta in the right panels (**B**, **D**, **F**, **H**, **J**, and **L**). For each stain, representative images are shown, followed by quantitative analyses of staining intensity performed by operators blinded to genotype and treatment. (**A** and **B**) Results of staining to detect protein carbonylation via oxy-immunohistochemistry (Oxy-IHC). Quantitation of Oxy-IHC staining shows similar increases in protein carbonylation in abdominal (**A**) and thoracic (**B**) aorta. (**C**–**H**) Similarly, stains for oxidized lipids (4-hydroxynonenal [HNE]), oxidized nucleic acids (8-hydoxyguanosine [8-OHG]), or tyrosine chlorination (3-chlorotyrosine [3-CT]) were increased in both thoracic and abdominal aorta in d-alanine–fed DAAO-TG^Tie2^ transgenic but not control mice. (**I**–**L**) Results of staining with antibodies directed against elastin to determine aortic wall thickness (**I** and **J**) and to quantitate elastin breaks (**K** and **L**) as an index of vessel wall integrity (69). Representative images are shown for each, as well as pooled results of quantitative histomorphometry measuring wall thickness and elastin breaks performed by blinded operators. *P < 0.05, **P < 0.01, ***P < 0.005, ****P < 0.001, 2-tailed Student’s *t* test. Markers of oxidative stress can be seen to increase in both abdominal and thoracic aorta following d-alanine feeding of DAAO-TG^Tie2^ transgenic mice (**A**–**H**). However, only the abdominal aorta shows wall thinning (**I**) and elastin breaks (**K**) following d-alanine feeding in DAAO-TG^Tie2^ transgenic mice. Scale bars: 100 μm. Pooled data are presented as means ± SEM of at least 3 independent experiments.

Having observed aneurysm formation in abdominal but not thoracic aorta (Figure 1) despite similar levels of transgene expression (Figure 2), we next explored whether these two aortic regions differed in the levels of oxidative stress induced by d-alanine feeding. Figure 3 shows the results of staining for a range of oxidation markers in sections of abdominal aorta versus thoracic aorta, quantitating the staining pattern seen for d-alanine–fed DAAO-TG^Tie2^ transgenic versus control mice. Figure 3, A and B, shows representative tissue immunostaining for detection of carbonylated proteins (24). Abdominal and thoracic aorta showed a similar degree of staining in d-alanine–fed DAAO-TG^Tie2^ mice (Figure 3, A and B), indicating similar increases in protein carbonylation throughout the vascular wall in abdominal and thoracic aortae — both of which showed significant increases in staining compared with d-alanine–fed wild-type controls. Figure 3, C and D, shows the results of immunostaining using antibodies against 4-hydroxynonenal (a marker of lipid peroxidation), which reveal a significant increase in lipid peroxidation in the vascular wall in both abdominal and thoracic aorta following d-alanine feeding of DAAO-TG^Tie2^ mice compared with controls — but again without significant differences between the abdominal and thoracic aorta. Similar results were seen with staining for oxidized nucleic acids: Figure 3, E and F, shows the results of immunostaining of abdominal and thoracic aortic sections using antibodies against 8-hydroxyguanosine, which reveal a marked increase in nucleic acid oxidation following d-alanine feeding of DAAO-TG^Tie2^ mice — yet again without significant differences between abdominal and thoracic aorta. Staining with antibodies against 3-chlorotyrosine (Figure 3, G and H) to detect protein oxidation yielded results similar to those seen with the other markers for oxidative stress shown above, indicating that generation of H_2_O_2_ in vascular endothelium in d-alanine–fed DAAO-TG^Tie2^ mice leads to similar increases of oxidation throughout the vascular wall for both abdominal and thoracic aorta. All these immunohistochemical markers for oxidation of biomolecules were significantly and similarly increased in abdominal and thoracic aorta from d-alanine–fed DAAO-TG^Tie2^ mice, indicating that the oxidative stress is similar in both vascular beds (Figure 3, C–H). By contrast, quantitative histomorphometry of abdominal and thoracic aorta showed significant decreases in wall thickness only in the abdominal aorta (Figure 3I), while the thickness of the thoracic aorta remained unchanged in DAAO-TG^Tie2^ mice versus controls (Figure 3J). We quantitated elastin breaks in aortic sections prepared from DAAO-TG^Tie2^ mice and found marked increases in elastin breaks in abdominal but not thoracic aorta following d-alanine feeding (Figure 3, K and L). Taken together, these observations indicate that the abdominal and thoracic aortae have similar levels of DAAO-TG^Tie2^ transgene expression and oxidative stress throughout the vascular wall, yet only the abdominal aorta undergoes aneurysm formation — along with thinning and disruption of the vascular wall. We therefore turned to proteomics analyses to identify the differential features of the vascular proteome in thoracic versus abdominal aorta in response to oxidative stress.

### Proteomics analysis of thoracic and abdominal aorta.

We treated DAAO-TG^Tie2^ and control mice with d-alanine for 3 months, and processed the tissues for proteomics analyses (25). Fold changes in protein abundance were calculated from peptide intensity values, as previously described (26). We detected about 1,100 proteins that had quantitatively significant peptide intensity values. In control animals, there was no difference in the proteomics profile between the thoracic and abdominal aorta. But proteomics analyses of thoracic and abdominal aorta isolated from d-alanine–fed DAAO-TG^Tie2^ mice (*n* = 3 for each condition) revealed an increase in 516 proteins in the abdominal aorta compared with the thoracic aorta and a decrease in about 500 proteins; 38 proteins were unchanged in abundance. We performed quantitative proteomics analyses using tandem mass tags (TMTs) (27), comparing abdominal aortic samples from DAAO-TG^Tie2^ (*n* = 3) and control mice (*n* = 3) after d-alanine feeding. We found no changes in the levels of antioxidant enzymes in these proteomics analyses nor any change in levels of nitric oxide synthases. The comparative proteomics findings were complemented by quantitative proteomics analyses (27), which identified about 7,000 annotated proteins in the abdominal aorta. These protein sets were ranked according to their quantification value and underwent further analyses to identify the pathways involved in the differential response to oxidative stress.

### Gene set enrichment analysis reveals processes involved in abdominal aneurysm formation.

**Figure 4:**
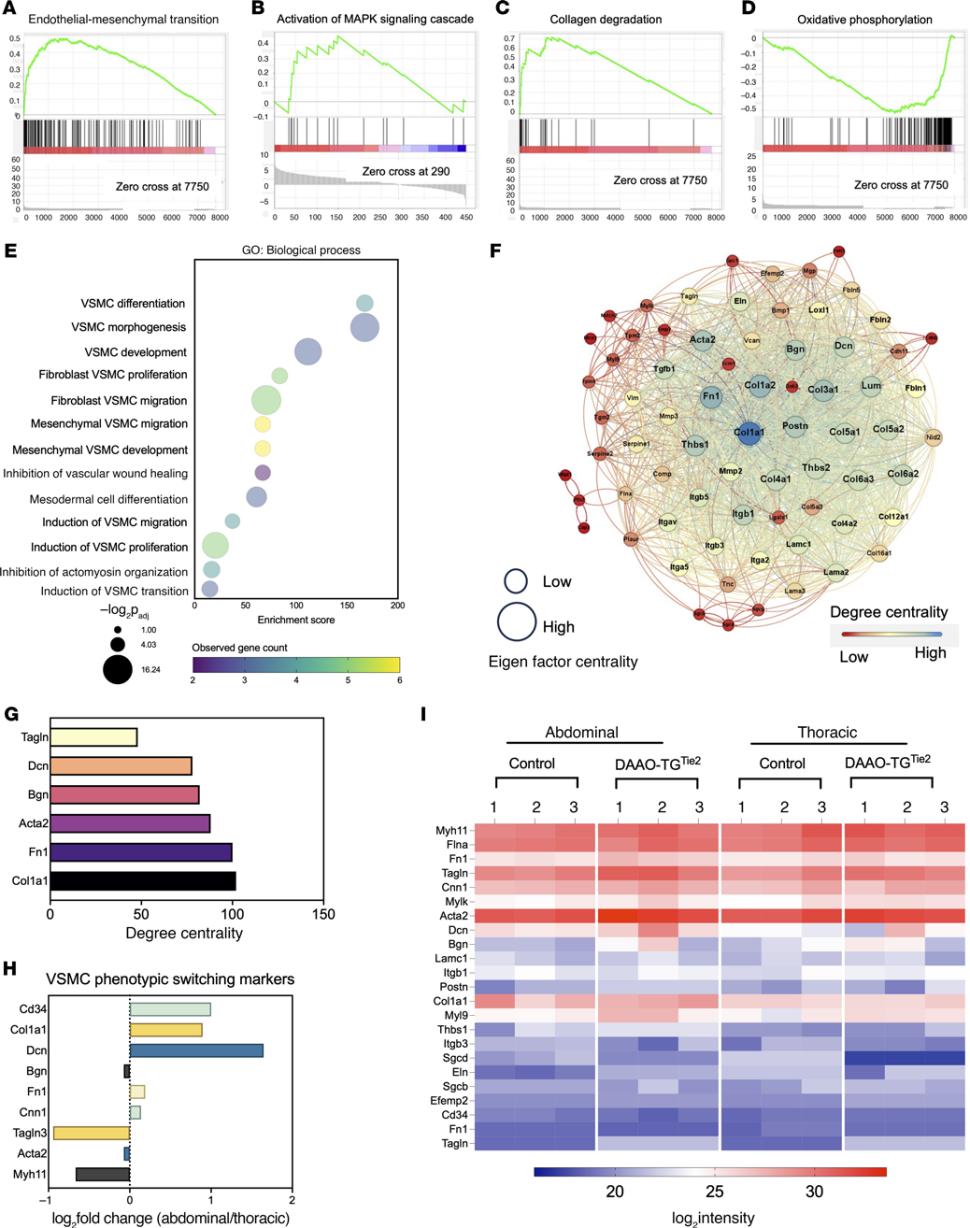
Proteomicsprotep analyses of thoracic and abdominal aorta following chemogenetic oxidative stress. (**A**–**D**) Results of GSEA of tandem mass tag (TMT) proteomics datasets from aortae isolated from d-alanine–fed DAAO-TG^Tie2^ transgenic or control mice subjected to chemogenetic oxidative stress for 3 months. Each GSEA (28) shown in **A**–**D** is characterized by FDR < 25% and *P* < 0.01. Aortae from d-alanine–fed DAAO-TG^Tie2^ transgenic mice exhibit significantly positive enrichment of pathways involved in EnMT (**A**); activation of MAPK signaling cascade (**B**); and collagen degradation (**C**). (**D**) Markedly negative enrichment of oxidative phosphorylation pathways in abdominal aorta in d-alanine–fed DAAO-TG^Tie2^ transgenic mice. (**E**) Bubble plot showing the top GO biological process enrichments from the EnMT reactome gene set (Supplemental Table 2). Enrichment score (on the abscissa) corresponds to the featured biological processes listed along the ordinate. Bubble size indicates –log_2_*P*_adj_, and bubble color indicates observed gene counts for each featured biological process. (**F**) Results of centrality analysis for enriched EnMT pathway proteins shown as an unweighted edge network with proteins shown as nodes and connections as edges. Node size was scaled on the Eigen factor value, and color gradient was assigned according to degree centrality scores of each node. Degree centrality is denoted by color as noted in the lookup table, with deep blue showing highest-order centrality and light green showing lowest-order centrality. Edge distances were assigned according to closeness centrality (40). (**G**) Bar graph presenting degree centrality of nodes involved in phenotypic switching characterized by having high degree centrality in the EnMT network shown in **A**. The abscissa shows degree centrality corresponding to gene names presented along the ordinate. (**H**) Bar graph of significant changes in VSMC phenotypic switching markers in thoracic versus abdominal aorta based on comparative proteomics data. The abscissa indicates log_2_ fold change of the ratio of abdominal to thoracic values for each marker that corresponds to the individual VSMC phenotypic switching markers shown along the ordinate. (**I**) Heatmap showing log_2_ intensity of all relevant proteins from the EnMT enrichment dataset (Supplemental Table 2) comparing protein intensity between abdominal and thoracic aorta from DAAO-TG^Tie2^ transgenic or control mice. Heatmap colors represent log_2_ intensity, with red indicating higher and blue indicating lower intensity. Data are representative of results of at least 3 independent experiments.

Quantified proteins were ranked using gene set enrichment analysis (GSEA) (28, 29) to identify the differentially enriched pathways and biological processes (BPs) in the abdominal aorta from d-alanine–treated DAAO-TG^Tie2^ animals compared with controls. These analyses revealed that the 4 most significantly enriched pathways were associated with aneurysm-related processes in the abdominal aorta. The most striking positive enrichments in the abdominal aorta of d-alanine–treated DAAO-TG^Tie2^ animals were for proteins that are associated either with endothelial-mesenchymal transition (EnMT) (Figure 4A) or with MAPK pathway activation (Figure 4B), which have previously been implicated in aneurysm formation (22–25). We also found that proteins involved in collagen degradation (Figure 4C) and oxidative phosphorylation (Figure 4D) showed significant positive and negative enrichment, respectively. These findings suggest an increase in processes involved in aneurysm formation (increased collagen degradation) accompanied by the development of redox imbalance (decreased oxidative phosphorylation) in abdominal aorta from d-alanine–treated DAAO-TG^Tie2^ mice.

### Gene Ontology biological process and network analyses implicate VSMC phenotypic switching.

The identification of EnMT (Figure 4A) by GSEA provides a critical clue in the biological effects of oxidative stress in the abdominal aorta. EnMT has been identified as a key determinant of vascular smooth muscle cell (VSMC) phenotypic switching and aortic aneurysm formation (30–34). We use the term “phenotypic switching” to refer generally to the transition from contractile to synthetic phenotypes, which can occur in response to diverse stimuli. We therefore performed Gene Ontology biological process (GO:BP) analysis (35) within the EnMT gene set to get further insight into the aneurysm-related biological processes in this model (the EnMT gene set is in Supplemental Table 2). GO:BP analysis identified enriched processes related to VSMC phenotypic switching: d-alanine treatment of DAAO-TG^Tie2^ animals caused a switch from the “contractile” vascular smooth muscle phenotype found in control aortae to a “synthetic” VSMC phenotype in which contractile proteins are lost and markers of fibrosis appear (36–38) (Figure 4E).

Phenotypic switching of VSMCs has been identified as a fundamental process in the pathogenesis of aortic aneurysms (38, 39). The enrichment of EnMT process identified in the proteomics studies (Figure 4A) indicates that abdominal aortic VSMCs in d-alanine–treated DAAO-TG^Tie2^ animals have undergone a phenotypic change. By contrast, proteomics analyses of thoracic aorta did not show any enrichment of EnMT pathways. There is complete concordance between the tissue phenotypes (Figures 2 and 3) and the proteomics signatures of abdominal and thoracic aorta from d-alanine–treated DAAO-TG^Tie2^ mice (Figures 3 and 4). To identify the principal regulators of the EnMT network (Figure 4F), we performed dynamic degree centrality analysis of the network (40, 41). Degree centrality values of different proteins in a network reflect the connectivity and importance of the different proteins that are expressed in a biological system, permitting the identification of the key regulatory hubs of that specific protein network. Our centrality measurements identified a subset of abdominal aorta proteins as the central regulatory nodes of the EnMT network in the aorta of d-alanine–treated DAAO-TG^Tie2^ mice. These proteins include collagen 1A1, α-smooth muscle actin, and other structural proteins seen in the VSMC extracellular matrix and in synthetic VSMCs. Of all these proteins, collagen 1A1 was identified as the single central hub of this network (Figure 4G and Supplemental Table 3).

After observing that VSMC phenotypic switching occurred in the abdominal aorta because of d-alanine treatment in DAAO-TG^Tie2^ mice (Figures 3 and 4), we compared the abdominal and thoracic proteomics datasets to determine whether VSMCs in both of these aortic regions underwent phenotypic switching. Comparative proteomics of abdominal and thoracic aorta indicated increased abundance of a mesenchymal VSMC marker (CD34) and fibroblast VSMC markers (Col1a1, Dcn, Fn1, Cnn1) in d-alanine–treated DAAO-TG^Tie2^ abdominal aorta but not in thoracic aorta (Figure 4H). We also observed a significant decrease in contractile proteins (α-SMA/Acta2, Myh11, Tgln3) only in the abdominal aorta of d-alanine–fed DAAO-TG^Tie2^ mice, indicating a shift from a “contractile” to a “synthetic” VSMC phenotype (42) (Figure 4H). Quantitation of the proteins in the EnMT dataset following GSEA analysis were plotted as a heatmap with reference to the abdominal and thoracic proteome dataset (Figure 4I and Supplemental Figure 7A). Taken together, these observations indicate that VSMCs of the abdominal aorta but not thoracic aorta undergo phenotypic switching in response to chronic oxidative stress. These regional findings in proteomics profiles comparing abdominal and thoracic aorta in d-alanine–treated DAAO-TG^Tie2^ animals exactly parallel the differences seen in the abdominal aorta when transgenic and control animals fed d-alanine are compared.

### Identification of JNK1 as the central regulator of VSMC phenotype switching.

**Figure 5:**
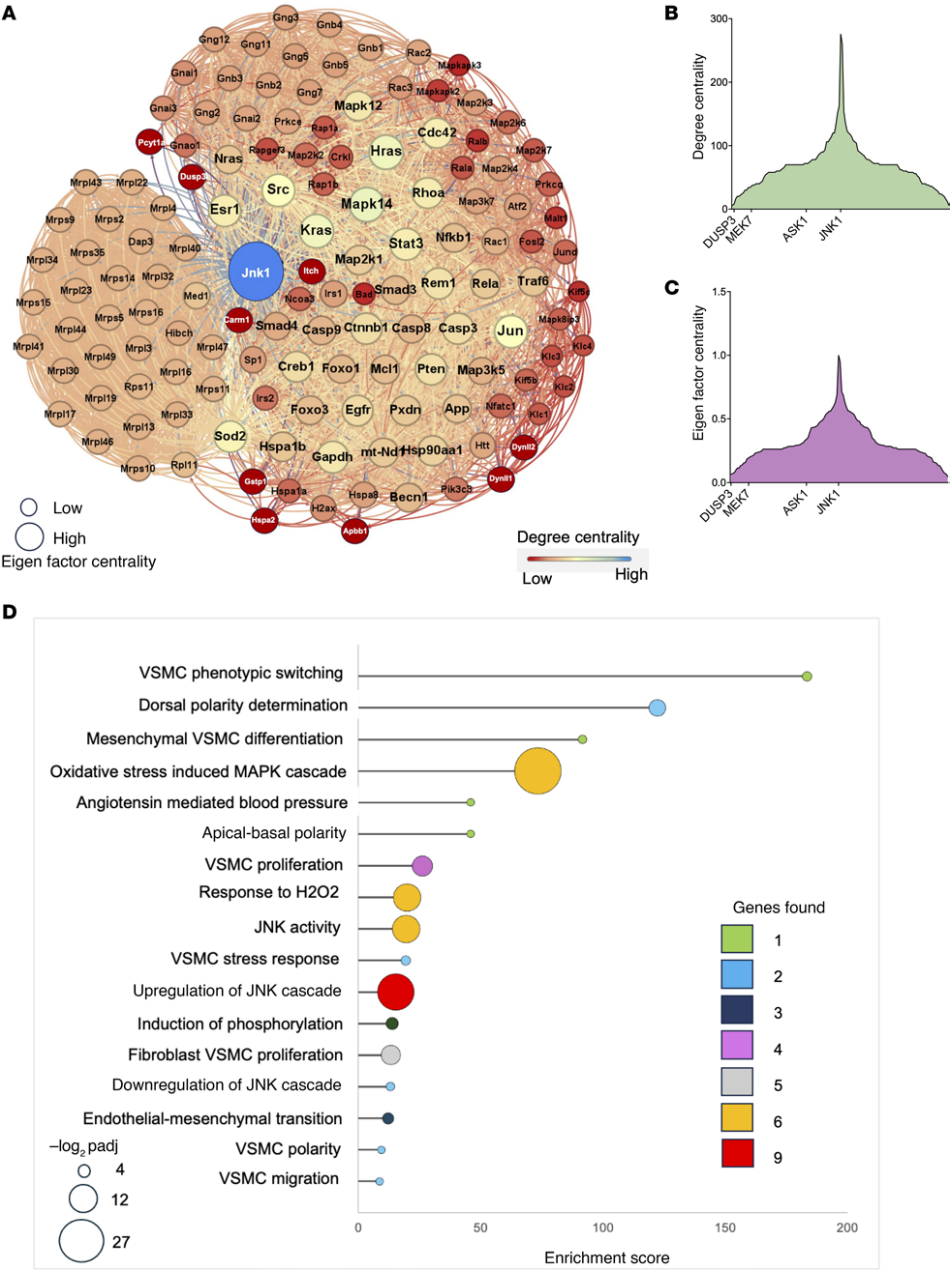
Quantitative proteomics analyses of abdominal aorta in d-alanine–treated DAAO-TG^Tie2^ and control mice. (**A**) The JNK1 protein network and connecting targets identified using quantitative proteomics (27) to compare abdominal aorta in d-alanine–treated DAAO-TG^Tie2^ transgenic and control mice. Node sizes were assigned according to the Eigen factor values, and the colors assigned according to the degree centrality (blue signifies highest centrality and red indicates lowest centrality). Edges are unweighted, but the distance between 2 nodes is assigned according to their closeness centrality values. (**B** and **C**) Gradient plots of the same data as shown in **A**. **B** shows selected MAPK cascade proteins along the abscissa, with the degree centrality of specific nodes quantitated along the ordinate. **C** is based on the same data as in **B**, but analyzed for Eigen factor centrality for selected MAPK cascade proteins, as shown on the ordinate. (**D**) Lollipop plot presenting the GO biological processes identified from the same set of proteins used to create **A**. The abscissa presents the enrichment score that corresponds to the enriched biological process listed on the ordinate. Color of the bubbles represents the number of genes identified in each process, and size of the bubbles represents the –log_2_*P*_adj_ values, as noted in the lookup table shown to the left of the plot. Data in this figure are based on the results of at least 3 independent experiments.

We next analyzed the quantitative abdominal aorta proteomics dataset using GO:BP analyses (35) and reactome pathway analyses to identify the most important proteins and interactions involved in the response to chemogenetic oxidative stress. These analyses identified the MAP kinase activation cascade (GO:0000165; *P* < 0.001) as the single most significantly enriched processes (Supplemental Tables 4 and 5). We focused on the MAP kinase subcluster from the quantitative proteomics dataset to identify central regulatory nodes. Degree centrality analysis of the MAP kinase subcluster indicated that the MAP kinase signaling protein JNK1 is the central hub of this network, implicating JNK1 as the central regulator of the MAP kinase pathway that is altered in the abdominal aorta of d-alanine–fed DAAO-TG^Tie2^ mice (Figure 5A). Analyses of centrality scores (degree centrality, Eigen factor centrality) (40, 41) for this network then identified the MAP kinase pathway signaling proteins ASK1, MEK7, and dual specificity phosphatase 3 (DUSP3) as the key determinants of JNK1 activity (Figure 5, B and C, and Supplemental Table 6). We again used GO:BP analysis to identify the critical biological processes enriched in the JNK1 network (Figure 5D). Our analysis identified a significant enrichment of processes related to oxidative stress; regulation of JNK cascade; and VSMC phenotypic switching (Figure 5D, Supplemental Figure 8A, and Supplemental Table 7). Many of these processes have been previously implicated in aneurysmal pathophysiology (17, 37, 38, 43), and our current proteomics findings identify a link between the JNK1 cascade and VSMC phenotypic switching.

**Figure 6:**
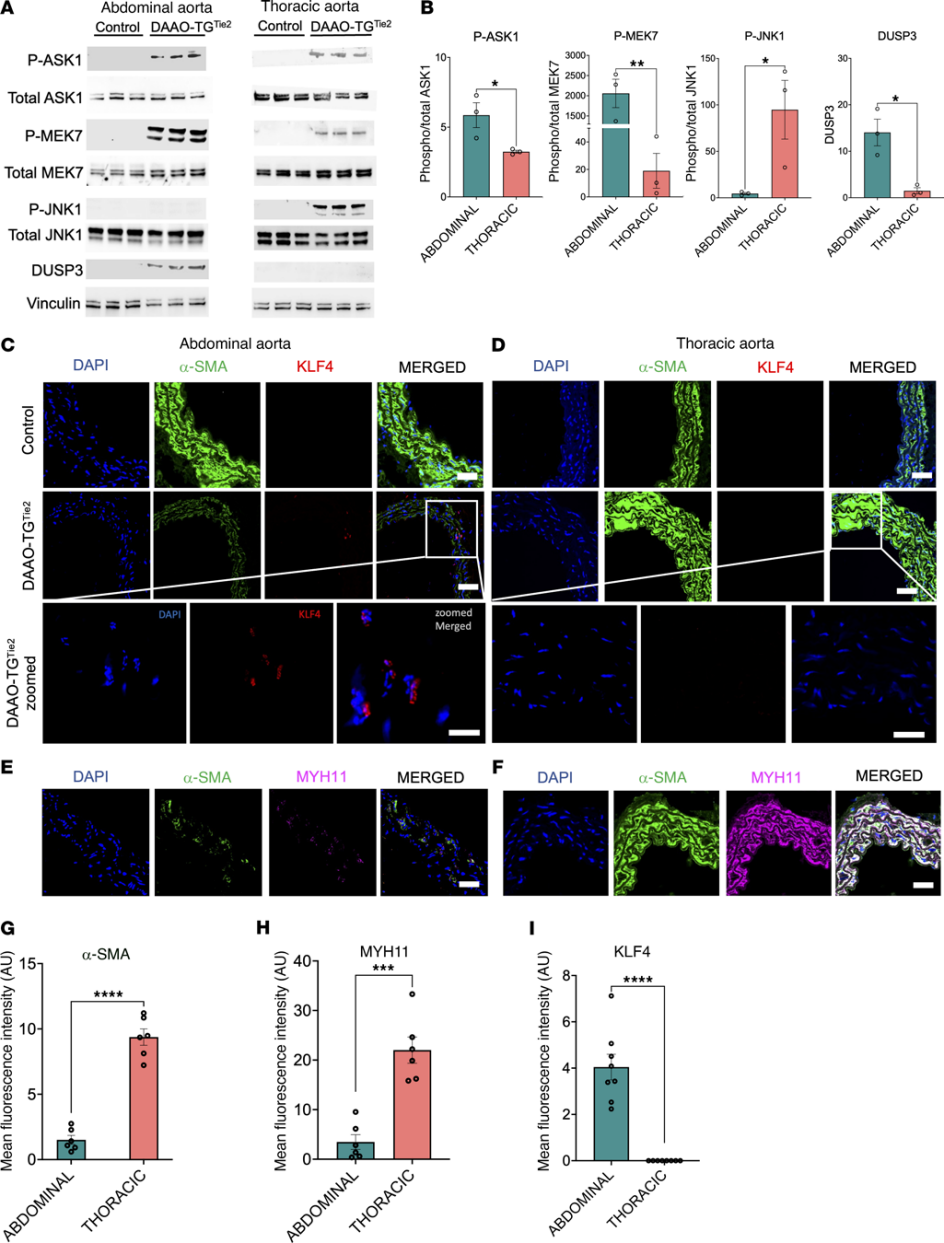
VSMC phenotypic switching in abdominal aorta but not in thoracic aorta. (**A**) Immunoblots probed with phospho-specific or total antibodies directed against 3 signaling proteins that are critically implicated in VSMC phenotypic switching (36, 42, 58) from the proteomics data: ASK1, MEK7, and JNK1. Each column presents immunoblots probing a single abdominal or thoracic aorta isolated from an individual d-alanine–treated DAAO-TG^Tie2^ transgenic or control mouse. (**B**) Bar graphs showing pooled densitometric quantification data from immunoblots for *n* = 3 mice for each experimental condition. *P < 0.05, **P < 0.01, 2-tailed Student’s *t* test, with vinculin used as a loading control. (**C**–**F**) Results of immunohistochemical staining (×40) in tissue sections isolated from abdominal (**C** and **E**) or thoracic (**D** and **F**) aorta from d-alanine–treated or control mice and probed with antibodies as shown. DAPI staining (nuclei) is shown in blue; staining with antibodies against α-SMA and KLF4 is shown in green and red, respectively. Scale bars: 100 μm. The bottom row of images in **C** and **D** presents higher-magnification (×60) views of the images shown in the row of photomicrographs presented in the row above; scale bars: 10 μm. (**E** and **F**) Results of immunohistochemical staining in abdominal (**E**) and thoracic (**F**) aorta sections in d-alanine–fed DAAO-TG^Tie2^ transgenic animals, with antibodies against the synthetic VSMC phenotypic switching markers α-SMA and MYH11 (42) shown in green and red, respectively. (**G**–**I**) Pooled data quantitating staining intensity for α-SMA (**G**), MYH11 (**H**), and KLF4 (**I**). ***P < 0.001, ****P < 0.0001, 2-tailed Student’s *t* test. Data are presented as means ± SEM of at least 3 independent experiments.

We hypothesized that oxidant-modulated signaling proteins might play a key role in activating the MAP kinase cascade, and we noted that the MAP kinase family member ASK1 (apoptosis signal–regulated kinase-1) undergoes autophosphorylation when it becomes oxidized (44–46). The abundance of key proteins involved in the GO:BP enrichments compared between abdominal aorta from control and d-alanine–treated DAAO-TG^Tie2^ mice indicated a significant and striking increase in the level of DUSP3 (Supplemental Figure 8B), a phosphoprotein phosphatase that dephosphorylates JNK1 and leads to a marked decrease in JNK1 activity (47–49). Since the protein levels of the 3 key MAPKs involved in JNK1 regulation (ASK1, MEK7, and JNK1) were unchanged (Supplemental Figure 8B), we speculated that the observed increase in DUSP3 abundance in aorta of d-alanine–treated DAAO-TG^Tie2^ mice (Supplemental Figure 8B) might lead to an increase in DUSP3-mediated dephosphorylation of JNK1, thereby leading to a decrease in JNK1 activity and the nuclear translocation of KLF4. We tested this hypothesis by exploring the pattern of phosphorylation of these MAP kinase signaling proteins in immunoblots probed with phospho-specific antibodies (Figure 6, A and B).

### JNK1 induces VSMC phenotypic switching in abdominal but not in thoracic aorta via the oxidant-modulated protein kinase ASK1.

Since the protein abundance level of the key MAP kinases of the cascade did not change, we probed immunoblots with phospho-specific antibodies against ASK1 and its downstream MAP kinases, which revealed major changes in the phosphorylation status of ASK1 and two other key signaling proteins involved in the MAP kinase signaling cascade (Figure 6A). We used phospho-specific antibodies against ASK1, MEK7, and JNK1 to probe immunoblots of abdominal and thoracic aorta from control and DAAO-TG^Tie2^ mice after 3 months of d-alanine treatment. Both ASK1 and MEK7 showed marked increases in phosphorylation in abdominal and thoracic aorta, while JNK1 showed a striking *decrease* in phosphorylation along with a significant increase in DUSP3 protein level in abdominal aorta compared with thoracic aorta (Figure 6B). These observations suggest that dephosphorylation of JNK1 by DUSP3 in the abdominal aorta leads to JNK1 inactivation, suggesting that JNK1 dephosphorylation by DUSP3 may be a critical determinant of aneurysm formation. JNK1 has been identified as a critical determinant of the nuclear localization of the transcription factor KLF4 (50–52). In turn, KLF4 has been identified as a critical determinant in VSMC phenotypic switching in aneurysm formation. JNK1 phosphorylation inhibits the translocation of KLF4 to the cell nucleus (50). Since JNK1 phosphorylation is decreased in abdominal (but not thoracic) aorta after d-alanine treatment of DAAO-TG^Tie2^ mice, we postulated that nuclear localization of KLF4 would be found in abdominal but not thoracic aorta following d-alanine feeding. Figure 6, C and D, shows the results of immunostaining for KLF4, and reveals that there was a striking increase in KLF4 (Figure 6I) in abdominal aorta of d-alanine–fed DAAO-TG^Tie2^ mice. We next probed for the abundance of two key KLF4 target genes, α-SMA and MYH11, which are important structural proteins in the vascular wall and are markers for contractile VSMCs (37, 42). We found that the abdominal aorta had significantly lower abundance of α-SMA and MYH11 compared with thoracic aorta in d-alanine–fed DAAO-TG^Tie2^ mice (Figure 6, E–H). The decrease in α-SMA and MYH11 in the abdominal aorta after d-alanine feeding of DAAO-TG^Tie2^ mice is consistent with a shift from a contractile to a synthetic VSMC phenotype because of increased KLF4, which itself is a consequence of dysregulated MAP kinase signaling in response to oxidative stress.

### Effects of DUSP3 inhibition on the vascular pathophenotype.

**Figure 7:**
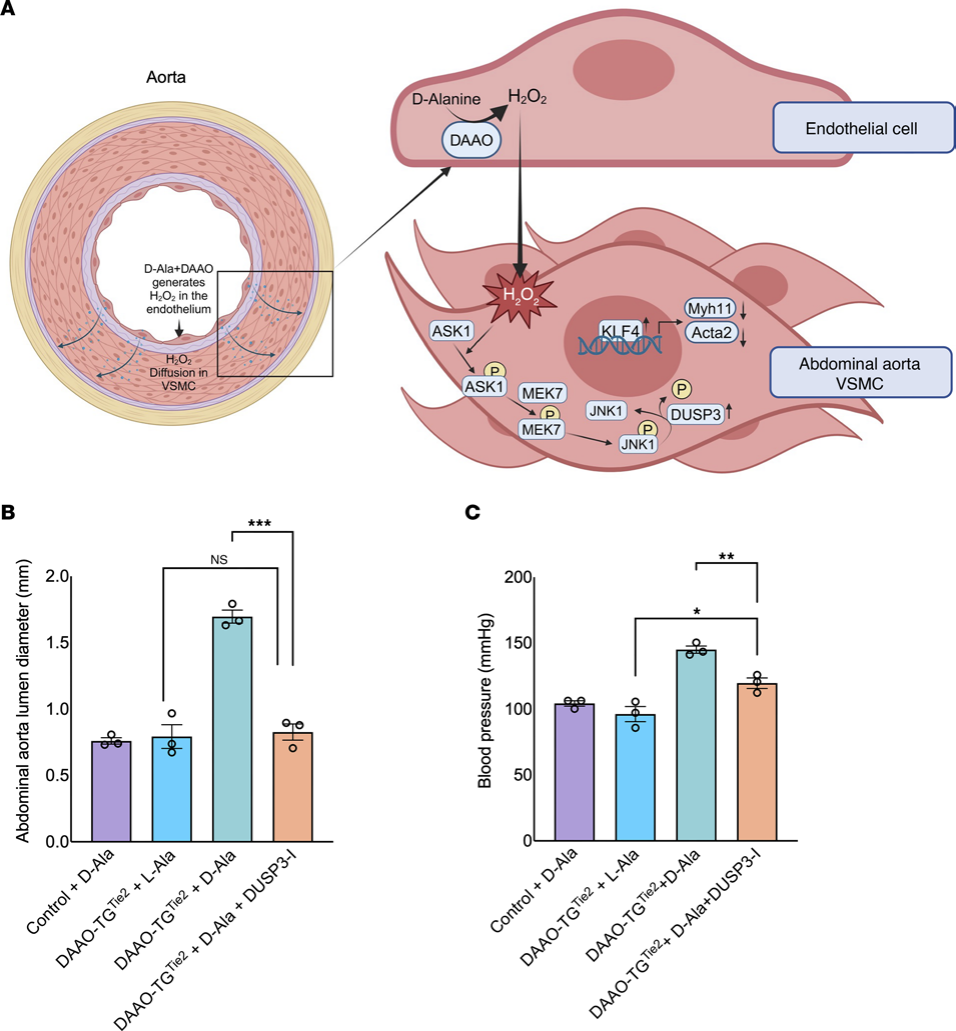
A key role for DUSP3 in abdominal aortic aneurysm formation caused by oxidative stress. (**A**) Left: Schematic showing that H_2_O_2_ generated by DAAO expressed in the vascular endothelium promotes oxidative stress throughout the vascular wall of d-alanine–fed DAAO-TG^Tie2^ transgenic mice (Figure 3). Right: Schematic of the signaling pathways initiated by the chemogenetic generation of H_2_O_2_ in vascular endothelial cells that lead ultimately to systemic hypertension and aortic aneurysm formation (Figure 1). H_2_O_2_ directly promotes ASK1 oxidation and autophosphorylation, which leads to the subsequent phosphorylation of MEK7 and JNK1 (52–57). The phosphoprotein phosphatase DUSP3 is present in abdominal but not thoracic VSMCs (Figure 6) and catalyzes the dephosphorylation of JNK1 (49), which is permissive for KLF4 translocation and leads to VSMC phenotypic switching (50). (**B** and **C**) Vascular phenotype of alanine-fed DAAO-TG^Tie2^ and control mice treated with the small-molecule DUSP3 inhibitor MLS-0437605 (DUSP3-I; 4 mg/kg/d by daily oral gavage) for 3 months. (**B**) Measurements of abdominal aorta diameter, showing that DUSP3-I treatment completely blocks the formation of aortic aneurysms in d-alanine–fed DAAO-TG^Tie2^ mice. (**C**) Systolic blood pressure measurements in control and DAAO-TG^Tie2^ transgenic mice after 3 months of drug plus d-alanine treatment, showing that DUSP3-I attenuates the increase in blood pressure seen in the d-alanine–fed DAAO-TG^Tie2^ mice that were not treated with DUSP3-I. There was a small but statistically significant increase in blood pressure comparing the L-alanine-fed DAAO-TGTie2 and the mice fed D-alanine plus DUSP3-I. *n* = 3 mice in each treatment group. *P < 0.05, **P < 0.01, ***P < 0.001, multiple 2-tailed Student’s *t* tests and ANOVA performed for multiple comparisons. Data are presented as means ± SEM of at least 3 independent experiments.

These studies have suggested a pathway leading from the generation of endothelial H_2_O_2_ in DAAO-TG^Tie2^ mice to yield a striking vascular pathophenotype characterized by systemic hypertension and abdominal aortic aneurysm formation (Figure 7A). A key role for the phosphoprotein phosphatase DUSP3 was suggested by both proteomics (Figure 5, B and C) and biochemical analyses (Figure 6, A and B). We sought further evidence for the role of DUSP3 by using an in vivo pharmacological approach to test the hypothesis that DUSP3 is a key determinant of the vascular pathophenotype seen after d-alanine feeding of DAAO-TG^Tie2^ mice. We administered the highly specific small-molecule DUSP3 inhibitor MLS-0437605 (53) by daily oral gavage (4 mg/kg/d) to DAAO-TG^Tie2^ and control mice at the initiation of d-alanine feeding and continued treatment for 3 months. As shown in Figure 7B, administration of the DUSP3 inhibitor completely blocked the development of abdominal aortic aneurysms in d-alanine–fed DAAO-TG^Tie2^ mice. The DUSP3 inhibitor also markedly attenuated the hypertension caused by d-alanine feeding in DAAO-TG^Tie2^ mice; there was still a small but statistically significant increase in blood pressure in the DUSP3-treated d-alanine–fed DAAO-TG^Tie2^ mice compared with the negative controls (Figure 7C).

## Discussion

These studies provide evidence that oxidative stress generated by recombinant d-amino acid oxidase expressed in vascular endothelial cells leads to abdominal aortic aneurysm formation, hypertension, and premature death in DAAO-TG^Tie2^ transgenic mice fed d-alanine (Figures 1 and 2).

Numerous previous reports in multiple animal models have provided evidence that oxidative stress is associated both with hypertension (1) and with the development of aortic aneurysms (8, 9). But the complexity and lack of specificity of these aneurysm models (8, 9) have made it difficult to establish that oxidative stress was itself causal. Here we have used chemogenetic approaches to establish that oxidative stress explicitly and specifically causes hypertension and aortic aneurysm formation. Confidence in this conclusion comes from rigorous genetic and treatment control experiments, which confirm that endothelial oxidative stress is necessary for this pathophenotype: neither aortic aneurysms nor hypertension develop in transgene-negative littermates fed d-alanine nor in DAAO-TG^Tie2^ transgenic mice fed l-alanine.

The formation of abdominal aortic aneurysms and the development of hypertension in DAAO-TG^Tie2^ transgenic mice are observed only after more than 2 months of d-alanine treatment, and this pathophenotype is not fully expressed until 3 months of treatment — soon after this point, all the d-alanine–fed transgenic animals die (Figure 1). We focused on characterizing the molecular and cellular features of the aorta at 3 months of d-alanine feeding, at which point the vascular pathophenotype was uniformly present in DAAO-TG^Tie2^ transgenic mice. We found that VSMCs throughout the abdominal as well as thoracic aorta showed evidence of protein carbonylation (Figure 3, A and B), lipid peroxidation (Figure 3, C and D), nucleic acid oxidation (Figure 3, E and F), and protein tyrosine chlorination (Figure 3, G and H). While expression of the DAAO transgene is limited to the vascular endothelium (Figure 2), markers of oxidative stress are seen throughout the vascular wall (Figure 3). Since H_2_O_2_ is a small lipophilic molecule, it is plausible that H_2_O_2_ generated by DAAO in endothelial cells diffuses throughout the vascular wall, causing oxidative modifications in VSMC proteins, lipids, and nucleic acids. It is also possible that oxidative damage limited to vascular endothelial cells leads to production of chemokines that attract inflammatory cells to the aortic wall, and it is these newly recruited cells that cause oxidative stress throughout the aortic wall. However, we found no increase in the prevalence of macrophages, neutrophils, dendritic cells, or T cells in the vascular wall of d-alanine–fed DAAO-TG^Tie2^ transgenic mice compared with controls (Supplemental Figure 5). It is still possible that inflammatory cells were present in the aortic wall earlier in the time course of d-alanine feeding but not at the study endpoint. However, bone marrow transplant experiments established that the vascular phenotype is a result of transgene expression in endothelial cells, not in hematopoietic cells. It is possible that other experimental approaches (e.g., single-cell RNA sequencing) could provide further clues into alterations of the cellular composition of the aorta in response to oxidative stress. In any event, our data indicate that activation of the DAAO transgene in vascular endothelial cells is the critical proximal cause of the pathophenotype.

Despite evidence for similar levels of oxidative stress throughout the abdominal and thoracic aortic walls of d-alanine–fed DAAO-TG^Tie2^ transgenic mice, only the abdominal aorta shows evidence of wall thinning (Figure 3, I and J) and elastin breaks (Figure 3, K and L), and only the abdominal aorta develops aneurysms leading to premature death (Figure 2). So while oxidative stress is necessary for development of the pathophenotype, *oxidative stress alone is not sufficient*: despite similar levels of transgene expression (Figure 2) and evidence for oxidative stress throughout the length of the aorta (Figure 3), only the abdominal aorta but not the thoracic aorta develops aneurysms. Despite the structural continuity of the aorta along its length, thoracic aorta and abdominal aorta arise from different embryological progenitors (18) and are subjected to different hemodynamic forces (54–56). Indeed, thoracic and abdominal aortic aneurysms in patients develop in response to different clinical and genetic risk factors, and patients who develop aortic aneurysms have different trajectories of disease progression and variable responses to therapy. The present studies provide proteomics and biochemical evidence indicating that differential signaling responses to vascular oxidants form the basis for differential development of aortic aneurysms in thoracic versus abdominal aorta despite similar levels of oxidative stress.

These studies have provided multiple lines of evidence indicating that phenotypic switching is taking place in VSMCs of the abdominal but not thoracic aorta in d-alanine–fed DAAO-TG^Tie2^ mice. Gene set enrichment analysis (GSEA) of comparative proteomics datasets (Figure 4) documents enrichment of aneurysm-related processes in abdominal aorta in response to oxidative stress. GSEA documents a significant positive enrichment of proteins involved in endothelial-mesenchymal transition (EnMT) (Figure 4A). EnMT has been implicated in aortic aneurysm formation via phenotypic switching of VSMCs. These findings establish a causal link between H_2_O_2_-mediated oxidative stress and vascular phenotypic switching in vivo.

The results of GSEA using comparative proteomics (Figure 4) are complemented and extended by quantitative proteomics analyses (Figure 5), which identified changes in the levels of specific proteins and their related connectivity networks between abdominal and thoracic aorta in d-alanine–fed DAAO-TG^Tie2^ mice. The MAP kinase signaling pathway, which has been previously implicated in the response to oxidative stress (57), was also identified in these studies. Quantitative proteomics analyses identified the MAP kinase signaling cascade involving JNK1, ASK1, MEK7, and DUSP3 in the pathway involved in VSMC phenotypic switching (Figure 5). The MAP kinase JNK1 was identified as the central node in this pathway, yet the protein abundance of JNK1, ASK1, and MEK7 was unchanged. These findings suggest that changes in the phosphorylation of MAP kinase signaling proteins might be a root cause of the observed phenotypic switching. We noted that the abundance of the phosphoprotein phosphatase DUSP3 is much greater in abdominal aorta in comparison with thoracic aorta (Figures 5 and 6). When we probed immunoblots of abdominal and thoracic aorta tissue isolated from d-alanine–fed DAAO-TG^Tie2^ mice with phospho-specific antibodies, we found a marked decrease in JNK1 phosphorylation in the abdominal aorta from d-alanine–fed DAAO-TG^Tie2^ mice. The dephosphorylation of JNK1 by the phosphoprotein phosphatase DUSP3 causes deactivation of JNK1 (49). We conclude that JNK1 is a key determinant of the phenotypic switch seen in abdominal aorta of d-alanine–fed DAAO-TG^Tie2^ mice, and this process is critically modulated by the phosphoprotein phosphatase DUSP3, which dephosphorylates and thereby inactivates JNK1. We next asked what are the molecular consequences of the dephosphorylation and inactivation of JNK1 in these cells, and how can this process be connected to VSMC phenotypic switching? Several lines of investigation implicate activation of the JNK1-modulated transcription factor KLF4 as the critical genetic determinant of the phenotypic switch in these cells.

KLF4 is a ubiquitous transcription factor that has been implicated in cellular dedifferentiation in a broad range of cell types. There are important connections between JNK1 and KLF4: The nuclear translocation and subsequent activation of KLF4 are suppressed by JNK1. Conversely, inhibition of JNK1 leads to an increase in KLF4 nuclear translocation and the activation of its transcriptional program. We used both immunoblot (Figure 6, A and B) and immunohistochemical (Figure 6, C–I) approaches to show that dephosphorylation of JNK1 is associated with a striking increase in KLF4 in the vascular wall in the abdominal aorta of d-alanine–fed DAAO-TG^Tie2^ mice. This increase in KLF4 is associated with significant decreases in the abundance of α-SMA and MYH11 (Figure 6, G–I), which are markers of the contractile VSMC phenotype (42), providing direct evidence of phenotypic switching in VSMCs (34, 36, 39, 42, 58).

**Figure 8:**
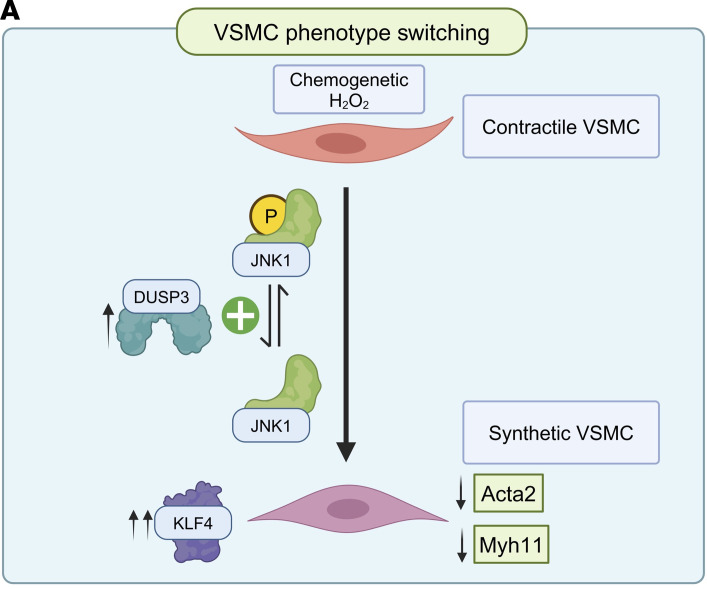
Schematic of key vascular signaling proteins connecting oxidative stress to phenotypic switching. This schematic focuses on the phenotypic switch from contractile to synthetic VSMCs in the abdominal but not thoracic aorta: dephosphorylation of phosphorylated JNK by the phosphoprotein phosphatase DUSP3 leads to nuclear translocation of the transcription factor KLF4, which causes decreased expression of Acta2 (α-SMA) and Myh11, leading to endothelial-mesenchymal transition and resulting in vascular wall thinning and aneurysm formation in the abdominal aorta.

These studies have used multiple experimental approaches to identify the proteins and pathways involved in this differential response to oxidative stress in abdominal versus thoracic aorta. These findings are summarized in a schematic (Figure 8) showing the pathways initiated by the chemogenetic generation of H_2_O_2_ in endothelial cells in the abdominal aorta of d-alanine–fed DAAO-TG^Tie2^ mice. Endothelium-derived H_2_O_2_ (generated by DAAO) activates the oxidant-modulated kinase ASK1 in VSMCs, promoting phosphorylation of the MAP kinase MEK7, which then phosphorylates JNK1 — which is the central determinant of phenotypic switching as identified in our proteomics analyses (Figure 5). The MAP kinase phosphatase DUSP3 is expressed in abdominal but not thoracic aorta (Figure 6). We postulate that DUSP3 promotes the dephosphorylation and consequent deactivation of the MAP kinase JNK1, which causes the nuclear translocation of the transcription factor KLF4, which promotes the phenotypic switch from contractile to synthetic VSMCs, leading to vascular wall thinning and aneurysm formation in the abdominal but not thoracic aorta.

Aortic aneurysms remain a major cause of morbidity and mortality worldwide, and current treatments and preventive strategies have limited efficacy. The present studies may provide a new incentive to explore the use of antioxidant supplements or drugs for the prevention or treatment of abdominal aortic aneurysms. The effects of antioxidants have not been explicitly studied in patients with abdominal aortic aneurysms (59), and it is plausible that administration of antioxidants might attenuate disease progression in selected patients with abdominal aortic aneurysms. The present studies have implicated the phosphoprotein phosphatase DUSP3 as a critical determinant of abdominal aortic aneurysm formation. The role of DUSP3 in redox metabolism is incompletely understood, but DUSP3-knockout mice show partial attenuation of kidney injury in a model of renal ischemia/reperfusion (60). Recent preclinical studies have explored roles for DUSP3 (61), and other phosphoprotein phosphatase inhibitors are being studied in a broad range of disease states (53, 62–64). Much remains to be learned about the cell-specific pathways that are deranged by reactive oxygen species, but we believe that chemogenetic approaches will continue to identify new pharmacological targets to combat the diverse disease states caused by oxidative stress.

## Methods

### Sex as a biological variable.

Our study examined both male and female animals, and similar findings are reported in both sexes.

### Mouse models.

All the experiments were carried out according to NIH guidelines for the care of laboratory mice, and all animal protocols were approved by the Brigham and Women’s Hospital Institutional Animal Care and Use Committee (protocol 2016N000278). Mice were housed in cages (<5 animals per cage) with regular chow diet (Purina Rodent Diet 5053) and continuous access to drinking water (with d-alanine or l-alanine, as indicated) on a 12-hour light/12-hour dark cycle. Room temperature was maintained at 21°C ± 2°C with 35% humidity. Studies were initiated when the animals were 8 weeks of age. Transgenic animals were developed and characterized as previously described (11). Littermates expressing the Cre recombinase but without the transgene (Cre^+^TG^−^) served as negative genetic controls. The primer sequences used for genotyping were: forward TTCCCTCGTGATCTGCAACTC and reverse CTTTAAGCCTGCCCAGAAGACT for Rosa26 wild-type; forward TTAATCCATATTGGCAGAACGAAAACG and reverse CAGGCTAAGTGCCTTCTCTACA for recognition of Cre recombinase; and forward GGGAGGTGTGGGAGGTTTT and reverse CTTTAAGCCTGCCCAGAAGACT for detection of the HyPer-DAAO transgene. All experimental mice were age- and sex-matched.

### Antibodies.

Primary antibodies used in these studies included GFP (anti-mouse, Cell Signaling Technology; clone 4B10; catalog 2955S); vinculin (anti-rabbit, Cell Signaling Technology; clone E1E9V; catalog 13901S); phospho-ASK1 (anti-rabbit phospho-Thr838; Invitrogen; catalog PA5-64541); ASK1 (anti-rabbit, Cell Signaling Technology; clone D11C9; catalog 8662S); Acta2 (anti-mouse, Novus Biologicals; catalog NBP2-22120); KLF4 (anti-rabbit, Proteintech; catalog 11880-1-AP); Myh11 (anti-rabbit, Proteintech; catalog 21404-1-AP); phospho-JNK (anti-rabbit, phospho-Thr183/Tyr185) (Cell Signaling Technology; clone 81E11; catalog 4668S); JNK (anti-rabbit, Cell Signaling Technology; catalog 9252S); MKK7 (anti-rabbit, Cell Signaling Technology; catalog 4172S); phospho-MKK7 (anti-rabbit, phosphor-Ser271/Thr275; Invitrogen; catalog MA5-28042); DUSP3 (anti-rabbit, Cell Signaling Technology; catalog 4752S); CD68 (anti-rabbit, Proteintech; catalog 257471-1-AP); Ly6V/Ly6G (anti-rabbit, Novus Biologicals; catalog NB600-1387); F4/80 (anti-rabbit, Novus Biologicals; catalog NB600-404SS); CD3E (anti-hamster, R&D Systems; catalog MAB484); CD45 (anti-rabbit, Cell Signaling Technology; clone 3F8Q; catalog 70257S); CD11c (anti-rabbit, Cell Signaling Technology; clone D1V9Y; catalog 97585S); 4-hydroxynonenal (anti-rabbit, Bioss Antibodies; catalog bs-6313R); 8-hydroxyguanosine (anti-rabbit, Bioss Antibodies; catalog bs-1278R); 4-chlorotyrosine (anti-rabbit, Hycult; catalog HP5002-20UG); 4-nitrotyrosine (anti-rabbit, Invitrogen; catalog A21285); and GAPDH (anti-rabbit, Cell Signaling Technology; clone 14C10; catalog 2118S).

The following secondary antibodies were used for immunofluorescence and immunoblot experiments: anti-rabbit IgG, HRP conjugated (Cell Signaling Technology; catalog 7074S); goat anti-rabbit Alexa Fluor 647 (Invitrogen; catalog A21245); goat anti-rabbit Alexa Fluor 594 (Invitrogen; catalog A11012); and goat anti-mouse Alexa Fluor 488 (Invitrogen; catalog A11001).

### Histology and quantitative histomorphometry.

All physiological and imaging analyses were performed by personnel blinded to genotype and/or treatment. Experimental and control animals were anesthetized with isoflurane and perfused with PBS and then with 4% paraformaldehyde. Abdominal and thoracic aorta were dissected out separately after perfusion and then fixed, embedded in paraffin or OCT compound, and sectioned. Slides were stained with hematoxylin and eosin, Van Gieson’s elastin stain (EVG), or Masson’s trichrome stain. Images were captured with an Axioskop microscope (Zeiss) equipped with a Excelis MPX-20C camera (Accu-Scope) and an Achroplan 10×/0.25 Ph1 as well as 40×/0.65 Ph1 objective (Zeiss). Images were captured and analyzed via Capta Vision software (Accu-Scope).

For oxy-immunohistochemistry (Oxy-IHC), fresh aorta was isolated from mice and fixed overnight with Methacarn (60% methanol, 30% chloroform, and 10% glacial acetic acid) at 4°C. Fixed tissues were paraffinized to prepare blocks and slides for sectioning (5 μm). Staining for carbonylated proteins was performed with the OxyIHC kit (Millipore), and images were quantified using ImageJ (NIH).

### Immunoblotting.

Thoracic and abdominal aortic tissues were collected after animal sacrifice, mechanically dissociated, and then lysed in RIPA lysis buffer (Boston BioProducts). After lysis, tissue samples were centrifuged at 13,400*g* for 15 minutes to precipitate tissue debris. Protein concentration was measured using BCA method (Thermo Fisher Scientific). Equal amounts of protein (20 μg) were mixed with 4× Laemmli buffer (Bio-Rad Laboratories), resolved on 10% polyacrylamide gels, and transferred to nitrocellulose membranes (Bio-Rad Laboratories). Membranes were washed with TBST (Tris-buffered saline with 0.1% Tween-20; Boston BioProducts) and blocked with 5% nonfat dry milk in TBST for 1 hour. Membranes were incubated at 4°C overnight with primary antibodies and then washed with TBST and incubated with HRP-labeled goat anti-rabbit immunoglobulin (Cell Signaling Technology). The membranes were washed (3 times for 5 minutes) with TBST and developed with enhanced chemiluminescence technique (Super Signal WestFemto, Thermo Fisher Scientific) and imaged with ChemiDoc MP Imaging System (Bio-Rad Laboratories).

### Immunofluorescence.

For immunofluorescence staining, mice were anesthetized, and fresh tissues were collected after intracardiac perfusion, fixed, and sectioned; slides were prepared in paraffin as well as OCT compound. The sections were deparaffinized, rehydrated, and washed in PBS. The sections were blocked with 2% BSA in PBS for 1 hour and stained with primary antibodies overnight at 4°C. The next day, sections were washed with PBS and incubated with secondary antibodies for 1 hour. Stained sections were observed under a Zeiss LSM700 confocal microscope in ×40 and ×60 oil immersion objectives, and images were captured in Zeiss Zen Black software. Images were analyzed via ImageJ.

Immunofluorescence images for oxidative stress markers were obtained on an inverted microscope (IX80, Olympus) equipped with a Lumen 200 Fluorescence Illumination System (Prior Scientific) and CCD camera (Hamamatsu) using a ×40 oil immersion objective (UPlanSApo, Olympus). Motorized filter wheels were controlled by a Sutter Lambda 10–3 controller (Sutter Instruments) and MetaMorph Imaging software version 7.10.5.476 (Molecular Devices LLC). Fluorescence of oxidative markers (4-hydroxynonenal, 3-chlorotyrosine, and 8-hydoxyguanosine) labeled with goat anti-rabbit Alexa Fluor 547 secondary antibody was captured using a dichroic filter (SpGold-B OMF, Semrock). Images were background-subtracted, and fluorescence intensities were quantified by the MetaMorph software.

For mouse-on-mouse immunofluorescence staining, the M.O.M. immunodetection kit (Vector Laboratories) was used, and the staining was carried out according to the manufacturer’s protocol.

### Blood pressure measurement.

Mice were acclimatized for 1 hour before measurement of blood pressure. The readings were taken on 3 consecutive days, and the readings of the last day were considered as the final reading and used for further analysis. Blood pressure was measured by tail cuff method (65) with a BP-2000 series II instrument (VisiTech Systems), and measurements were recorded with a BP-2000 blood pressure analyzer. The observers were blinded to genotype and to treatment.

### H_2_O_2_ quantification in aortic tissues using the Amplex Red assay.

Thoracic and abdominal aortae were isolated from d-alanine–fed (3 months, 0.75 M) or untreated DAAO-TG^Tie2^ mice. The aortae were cleared of any associated fat or tissue, cut into 2 mm rings, and incubated for 45 minutes in Krebs-Ringer phosphate-glucose buffer (pH 7.4) at 37°C. H_2_O_2_ was measured using the Amplex Red assay following the manufacturer’s protocols (Life Technologies). Levels of H_2_O_2_ were calculated based on a contemporaneous H_2_O_2_ standard curve and the results presented as picomoles per minute per aortic ring.

### DUSP3 inhibitor treatment.

DUSP3 inhibitor (DUSP3-I; MLS-0437605) was from MedChem Express. Four groups of mice were set up for the experiment. A genetic control consisted of Cre^+^, transgene-negative mice fed with d-alanine, and a treatment control group consisted of DAAO-TG^Tie2^ transgenic mice fed l-alanine. The experimental groups were DAAO-TG^Tie2^ transgenic mice, and both were provided with d-alanine in their drinking water; in one group DUSP3-I was administered by daily oral gavage at a dose of 4 mg/kg/d. There were 3 mice in each group, and blood pressure and vascular sonography measurements were obtained by observers blinded to genotype and to treatment.

### Vascular ultrasonography.

Vascular ultrasonography was performed as previously described (10) using a Visual Sonics F2 system equipped with a UHF57x probe. Treated control and transgenic mice were anesthetized with isoflurane to sustain heart rate above 450 bpm. Standard echocardiographic images of transverse and longitudinal views of abdominal and thoracic aorta were obtained. Images were analyzed with Vevo LAB software (v3.1.1, FUJIFILM VisualSonics). The sonographer and analyzer were blinded to the experimental treatment and/or genotype. All sonograph images were taken in the diastolic state of the aorta.

### Bone marrow transplantation.

Wild-type (*n* = 3) and DAAO-TG^Tie2^ (*n* = 3) mice were γ-irradiated (11 Gy in 2 exposures, 5.5 Gy each) for 15 minutes. DAAO-TG^Tie2^ bone marrow (*n* = 3) was injected into irradiated wild-type mice, and wild-type bone marrow (*n* = 3) was injected into irradiated DAAO-TG^Tie2^ mice via tail vein injection. The mice were provided with drinking water containing 0.75 M D-alanine along with antibiotics for 3 months. Aortic sonography was performed on the mice every 2 weeks for 3 months to observe aneurysm formation.

### Proteomics analyses of aortic tissues.

Fresh thoracic and abdominal aorta tissues were collected separately from control (*n* = 3) and transgenic mice (*n* = 3) and homogenized in RIPA lysis buffer. Total protein was TCA-precipitated and resolubilized in RapiGest SF (Waters). Resolubilized proteins were reduced with DTT (10 mM, 30 minutes, 80°C) and alkylated with iodoacetamide (20 mM, 30 minutes at room temperature). Two milliliters of modified sequencing-grade trypsin (20 ng/mL; Promega) was added to each sample, and the samples were placed in a 37°C water bath overnight. Before proteomics analyses, samples were acidified by addition of 20 μL 20% formic acid solution and then desalted using a C18 Stage tip (Thermo Fisher Scientific).

Liquid chromatography–mass spectrometry (LC-MS) analyses followed established protocols (11, 25). On the day of analysis, the samples were reconstituted in 10 μL of HPLC solvent A (66). A reverse-phase HPLC capillary column was created by packing of C18 spherical silica beads into a silica capillary. Each sample was loaded via a FAMOS autosampler (LC Packings). A gradient was formed, and peptides were eluted with increasing concentrations of solvent B (97.5% acetonitrile, 0.1% formic acid). Eluted peptides were subjected to electrospray ionization and then passed through an LTQ Orbitrap Velos Elite ion trap mass spectrometer (Thermo Fisher Scientific). Peptides were detected, isolated, and fragmented to produce a tandem mass spectrum of specific fragment ions for each peptide. Peptide sequences were determined by matching of protein databases with the acquired fragmentation pattern by the software program Sequest (Thermo Fisher Scientific). All databases include a reversed version of all the sequences, and the data were filtered to reduce the peptide false discovery rate (FDR) to 1%–2%.

The label-free quantification values of the annotated proteins were normalized by log_2_ transformation. Pairwise comparison between groups was done by 2-tailed *t* test and 1-way ANOVA using GraphPad Prism 9. Differences according to Benjamini-Hochberg adjusted *P* value less than 0.05 were considered significant.

### Tandem mass tag (TMT) labeling and quantitative proteomics analyses.

Samples for quantitative protein analysis were prepared as previously described (67). Proteins were extracted from aortic tissues using urea lysis buffer (Roche). After tissue lysis, 25 μg of each protein was reduced with 5 mM TCEP. Cysteine residues were alkylated using 10 mM iodoacetamide. Excess iodoacetamide was quenched with 10 mM DTT. A buffer exchange was carried out using a modified solvent precipitation 3 (SP3) protocol (66). Samples were digested overnight at room temperature. The next morning, trypsin was added to each sample and incubated for 6 hours at 37°C. Acetonitrile was added to each sample to a final concentration of approximately 33%. Each sample was labeled in the presence of SP3 beads, with approximately 62.5 μg of TMTpro reagents (Thermo Fisher Scientific), and then desalted via C18 Stage tips (Thermo Fisher Scientific) and redissolved in 5% formic acid/5% acetonitrile for LC-MS3 analysis via an Orbitrap mass spectrometer.

Raw files were converted to mzXML, and monoisotopic peaks were reassigned using Monocle (68). Searches were performed using the Comet search algorithm (https://comet-ms.sourceforge.net/) against a mouse database downloaded from UniProt in May 2021. We used a 50 ppm precursor ion tolerance, 1.0005 fragment ion tolerance, and 0.4 fragment bin offset for MS2 scans collected in the ion trap. TMTpro on lysine residues and peptide N-termini (+304.2071 Da) and carbamidomethylation of cysteine residues (+57.0215 Da) were set as static modifications, while oxidation of methionine residues (+15.9949 Da) was set as a variable modification. Each run was filtered separately to 1% FDR on the peptide-spectrum match level, as previously described (27, 67).

### Proteomics data analysis.

Gene set enrichment analysis (GSEA) of proteomics datasets of comparative abdominal-thoracic proteome and quantitative abdominal aorta proteome was performed on a preranked annotated protein set as previously described (29) against reference Molecular Signatures Database (MSigDB). *P* values less than 0.05 and FDR values less than 0.25 were considered significant for the analysis (28).

Networks were created with the R package igraph, and degree centrality analysis was carried out with the Gephi-supported R package rgexf. Networks were visualized with Cytoscape and R. The R package go.db was used for GO:BP enrichment analysis of annotated proteome with DAVID-based annotated GO terms (*Mus musculus*) as reference. Pathway analysis of annotated proteins was performed using the R package clusterProfiler against the mouse Reactome pathway database. Heatmaps were created using the R package Complex Heatmap (version 2.10.0). Bar graphs and histograms were built in GraphPad Prism 9.

### Statistics.

Statistical analysis for between-group comparisons was performed using 2-tailed Student’s *t* test (for 2-group comparisons) or 2-way ANOVA with appropriate post-testing (for comparisons between 3 or more groups). Data values are presented as individual data points and expressed as means ± standard error of the mean (SEM). Individual statistical tests are described in the corresponding figure legends. *P* values less than 0.05 were considered statistically significant. Equal numbers of male and female mice were studied. All physiological and imaging studies were performed and analyzed by scientists blinded to genotype and treatment. Statistical analyses were performed using GraphPad Prism 9.0 (GraphPad Software).

### Study approval.

All the experiments were carried out according to NIH guidelines for the care of laboratory mice, and all animal protocols were approved by the Brigham and Women’s Hospital Institutional Animal Care and Use Committee (protocol 2016N000278).

### Data availability.

All data are available in the article and its supplemental material and Supporting Data Values file. Raw proteomics data were submitted to the Proteomics Identifications Database (PRIDE) repository (PXD060700, PXD060737).

## Author contributions

AAD, MWW, FS, SY, AP, TD, and TAC designed and performed experiments, and were involved in experimental design and interpretation along with TM. TM and AAD wrote the manuscript.

## Supplementary Material

Supplemental data

Unedited blot and gel images

Supplemental table 2

Supplemental table 3

Supplemental table 4

Supplemental table 5

Supplemental table 6

Supplemental video 1

Supplemental video 2

Supporting data values

## Version 1 03/18/2025

In-Press Preview

## Version 2 05/01/2025

Electronic publication
