# Supplementary material for: Differential aortic aneurysm formation provoked by chemogenetic oxidative stress
Source: J Clin Invest. 2025 Mar 18;135(9):e188743. doi: 10.1172/JCI188743 (PMC12043099; doi:10.1172/JCI188743)
Supplement: Supplemental data [file jci-135-188743-s165.pdf]

Supplemental Materials

Supplemental Materials

Supplemental Tables 1-7

Supplemental Figures 1-8

Supplemental Videos 1-2

Supplemental Tables

Group	Sex	No. of animals	Average infrarenal aorta diameter (mm+/- SD)
Control	M	6	0.554+/- 0.03
	F	6	0.541+/- 0.04
DAAO-TG^{Tie2}	M	6	1.008 +/- 0.09
	F	6	1.129+/- 0.14

Supplemental Table 1. No intra-group variations observed in infrarenal aortic diameter (aortic sonogram) between male and female Control and DAAO-TG^{Tie2} mice after D-alanine treatment

Supplemental tables submitted as excel tables

Supplemental Table 2: List of EnMT gene sets obtained from GSEA analysis (Figure 4A) showing enrichment score of the gene sets.

Supplemental Table 3. Detailed centrality analysis scores for each node (Proteins) for EnMT network (Figure 4F).

Supplemental Table 4. Detailed results of GO: Biological Process analysis from quantitative abdominal aorta proteome data. Relevant pathways are highlighted in yellow.

Supplemental Table 5. Detailed results of Reactome pathway analysis from quantitative abdominal aorta proteome data.

Supplemental Table 6. Detailed centrality analysis scores for each node (Proteins) for JNK1 network (Figure 5A).

Enriched Biological Processes	Proteins
VSMC migration	Rapgef3, Src
Cell polarity	Crkl, Hsp90aa1
Endothelial-Mesenchymal transition	Smad3, Smad4, Ctnnb1
Negative regulation of JNK1 cascade	Dusp3, Itch
Fibroblast VSMC differentiation	Fosl2, Jun, Esr1,Egfr,Nras
Positive regulation of JNK1 cascade	Map2k7(MEK7), Map3k5(ASK1),Map3k7,Mapk8ip3,Hras,Cdc42,Traf6,App
Oxidative stress induced MAPK cascade	Map2k6, Map2k7(MEK7), Map3k5(ASK1),Map3k7,Mapk8(JNK1),Mapk14
VSMC phenotypic switching and response to H2O2	Sod2, Bad, Src,Casp3,Jun,Mapk8(JNK1)
Mesenchymal VSMC differentiation and regulation of systemic blood pressure	Rhoa,Ctnnb1

Supplemental Table 7. List of proteins corresponding to enriched GO: Biological Processes from quantitative abdominal aorta proteome analysis shown in Figure 5D.

Supplemental Figures

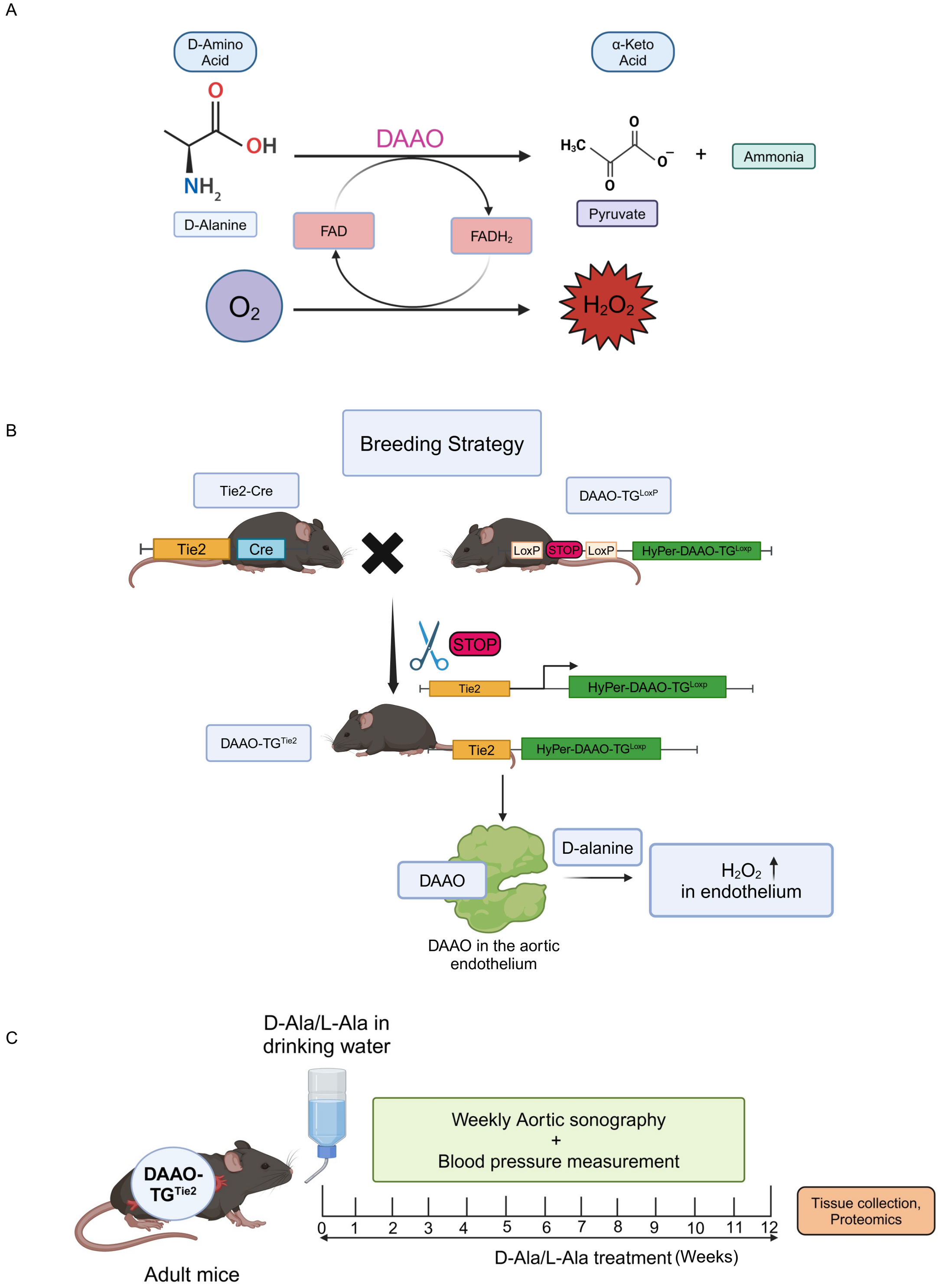

Supplemental Figure 1: Summary of the DAAO catalytic scheme and the strategy for generation and characterization of DAAO-TG^{Tie2} transgenic mice

Panel A shows the catalytic scheme for the oxidation of D-amino acids by D-amino acid oxidase (DAAO), which generates equimolar amounts of the corresponding a-keto acid, hydrogen peroxide (H_2O_2) and ammonia (NH_3). When D-alanine is the substrate, pyruvate is the keto acid product. The intracellular concentrations of pyruvate and ammonia are orders of magnitude higher than hydrogen peroxide(1) ; after activation of recombinant DAAO by addition of D-alanine, the relative increase in intracellular pyruvate and ammonia are nominal, while the intracellular concentration of hydrogen peroxide increases ~2-fold(1).

Panel B shows the breeding strategy we used to generate the DAAO-TG^{Tie2} transgenic mouse line. A transgenic mouse line that expresses Cre recombinase under control of the endothelial cell-specific Tie2 promoter (Jackson Labs) was crossed with DAAO-TG^{LoxP} transgenic mice(1) in which a stop codon flanked by LoxP recombination sites had been cloned into the DAAO-HyPer transgene coding sequence. Offspring are screened by PCR to identify founder lines containing the Tie2 promoter in continuity with the DAAO transgene, and functional expression of the transgene was validated in vascular endothelium(2).

In **Panel C** two different controls are used: DAAO-TG^{Tie2} transgene-positive littermates are fed D-alanine with L-alanine used as a treatment control, or Cre-positive/transgene-negative (Cre^{+}/TG^{-}) littermates fed D-alanine are used as a genetic control for the D-alanine-fed DAAO-TG^{Tie2} mice.

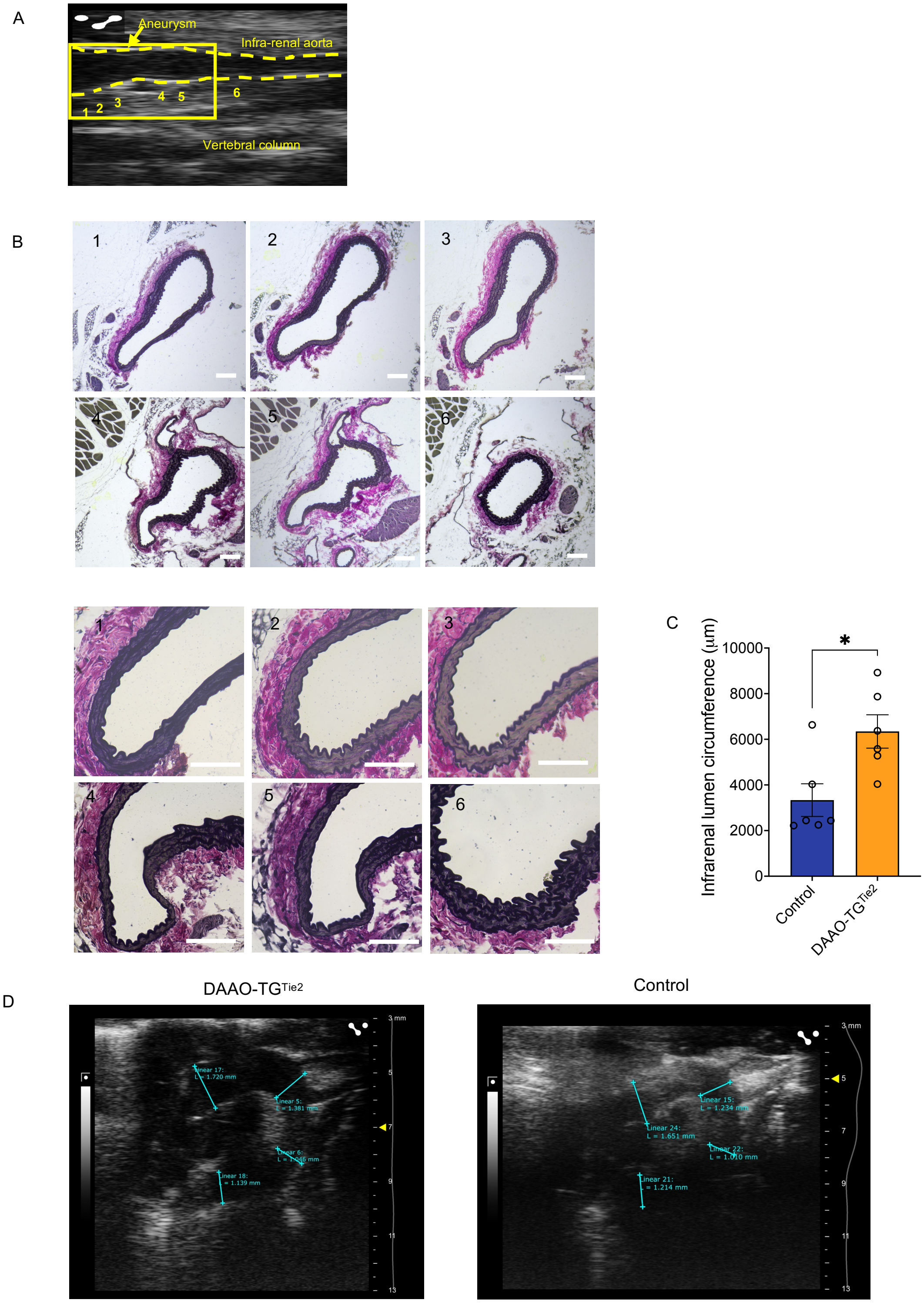
A
Aneurysm
Infra-renal aorta
1 2 3 4 5 6
Vertebral column
B
1
2
3
4
5
6
C
Infrarenal lumen circumference (μm)
10000
8000
6000
4000
2000
0
*
Control
DAAO-TG^Tie2
D
DAAO-TG^Tie2
Control

Supplemental Figure 2: Imaging of abdominal and thoracic aorta from D-alanine-fed DAAO-TG^{Tie2} and control mice

The aortic sonography (zoomed) image (obtained in diastole) show the presence of aneurysms in infrarenal abdominal aorta isolated from DAAO-TG^{Tie2} (**Panel A**) mice that were fed D-alanine for 3 months. After tissue fixation of the aorta, transverse aortic sections were stained for elastin ("Elastin") from the same infra-renal portion of abdominal aorta as shown in **Panel A**. **Panel B** shows images (slices at numbers 1to 6 in panel A) of corresponding (sonographed) aneurysmal portion of infrarenal abdominal aorta stained for elastin. The scale bars indicate 40 µm for lower magnification and 10µm for lower magnification. **Panel C** show average infrarenal lumen circumference of control (n=6) and DAAO-TG^{Tie2} (n=6) mice measured from histological sections at the same infrarenal location. **Panel D** show aortic sonography of DAAO-TG^{Tie2} and control thoracic aorta (corresponding to images presented in Figure 2E and 2F) with measurements at that aortic root, ascending aorta and descending aorta (all imaging at diastole). Data shown here represents mean ± SEM of at least 3 independent experiments.

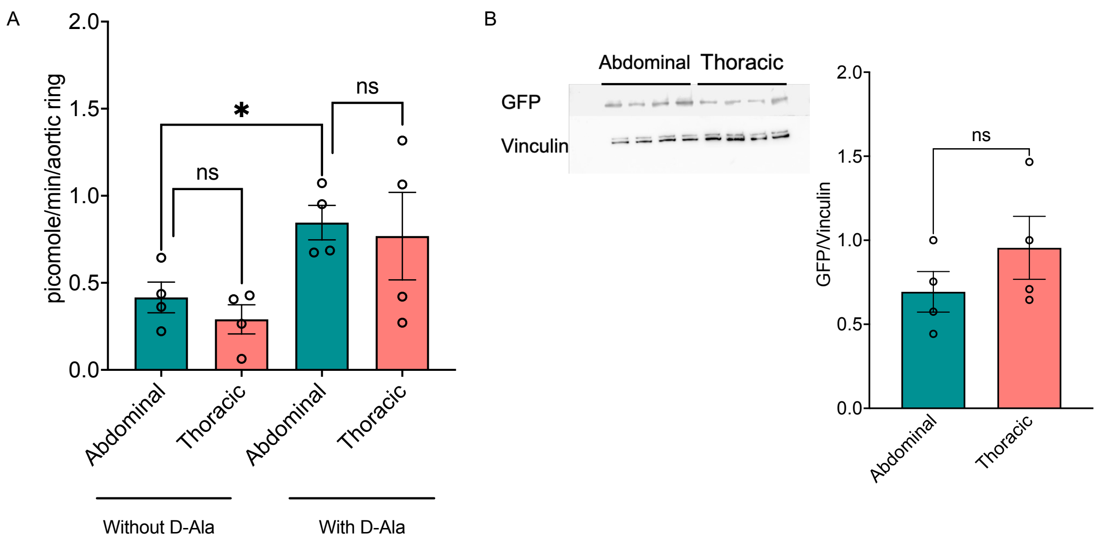

Supplemental Figure 3: Quantitation of hydrogen peroxide levels and transgene expression in abdominal and thoracic aorta.

Panel A shows the results of Amplex Red assays in abdominal and thoracic aorta segments isolated from untreated or D-alanine-treated (0.75 M D-alanine for 3 months) DAAO-TG^{Tie2} mice, as indicated in the graph. Freshly-isolated aorta tissues were cut transversely into 2 mm-long rings from the thoracic or abdominal aorta, and then incubated for 45 minutes at 37°C in Krebs Ringers Phosphate Glucose assay buffer containing Amplex Red and quantitated for fluorescence in a plate reader (Varioskan) according to the manufacturer's instructions (Life Technologies). * indicates $p<0.05$(Student's t test), ns indicates no statistically significant difference.

Panel B shows the results of immunoblots prepared from thoracic or abdominal aorta isolated from individual DAAO-TG^{Tie2} mice and probed with antibodies against GFP (which detects the YFP present in the transgene) or Vinculin (as a loading control). The bar graph presents the results of pooled data from 4 individual mice showing the ratio of GFP to Vinculin band intensity; ns

indicates no significant difference. Data shown here represents mean ± SEM of at least 3 independent experiments.

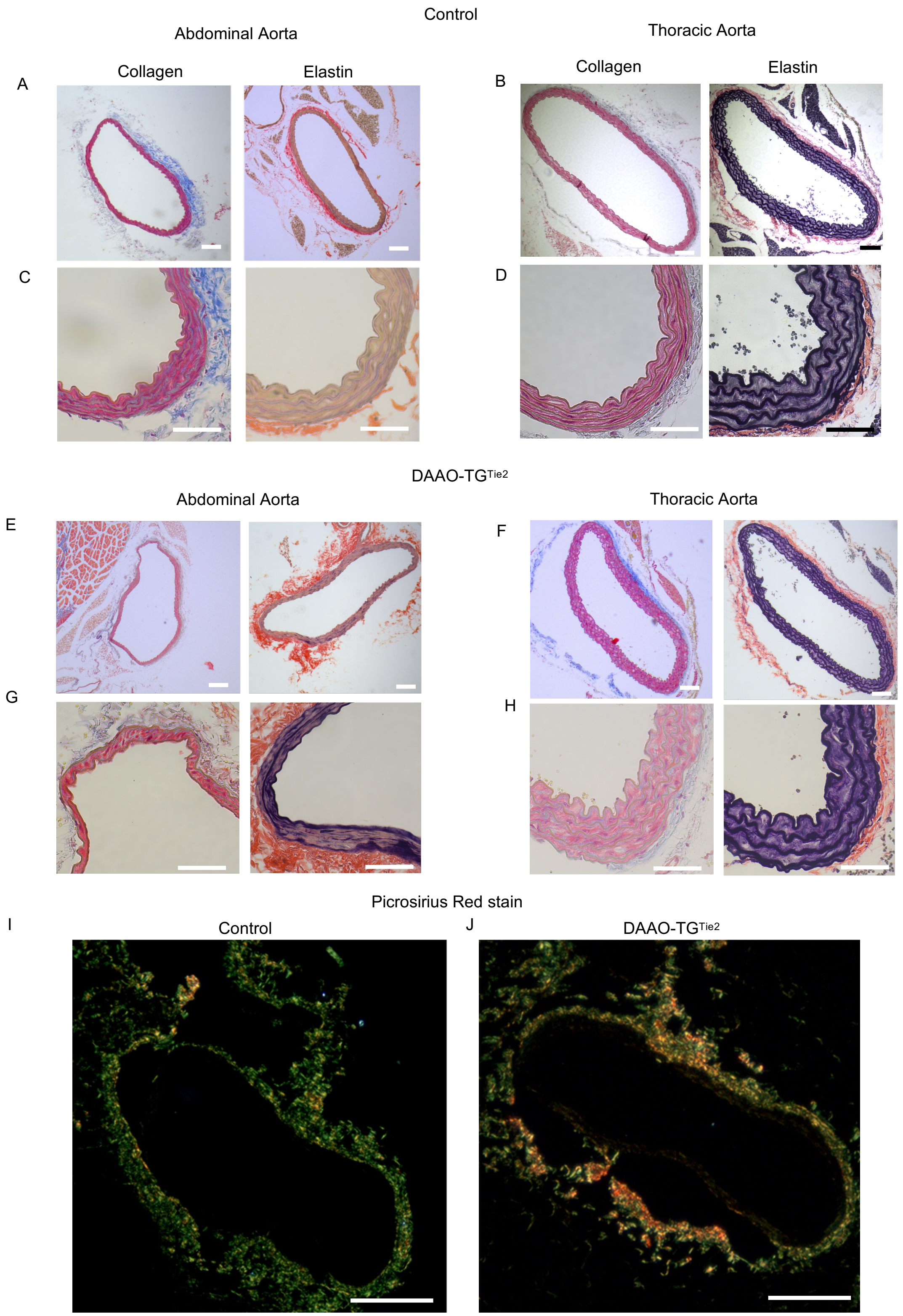
Control
Abdominal Aorta
Thoracic Aorta
Collagen
Elastin
Collagen
Elastin
A
B
C
D
DAAO-TG^{Tie2}
Abdominal Aorta
Thoracic Aorta
E
F
G
H
Picrosirius Red stain
I
Control
J
DAAO-TG^{Tie2}

97

98

Supplemental Figure 4: Histopathological staining of abdominal and thoracic aorta from D-alanine-fed DAAO-TG^{Tie2} and control mice

These photomicrographs show the results of histopathological staining in infrarenal abdominal and descending thoracic aortae isolated from control (**Panels A and B**:10X and Panels **C** and **D**:40X) or DAAO-TG^{Tie2} (**Panels E and F**:10X and **Panels G and H**: 40X) mice that were fed D-alanine for 3 months. After tissue fixation, transverse aortic sections were stained either with Masson's trichrome stain for collagen ("Collagen") or with Van Gieson's stain for elastin ("Elastin"). Van Gieson's stained thoracic aorta sections of control and DAAO-TG^{Tie2} (10X and 40X) are the same images as of Figure2F.The left panels show images of abdominal aorta, and the right panels show results from thoracic aorta. The scale bars show 100 µm. Shown below in **Panels I and J** are representative polarized light microscopy images of transverse abdominal aorta sections from D-alanine-fed control and DAAO-TG^{Tie2} mice (n=3 for each group) stained with Picrosirius Red to detect type I collagen (orange-red) and type III collagen (green).

Supplemental Figure 5

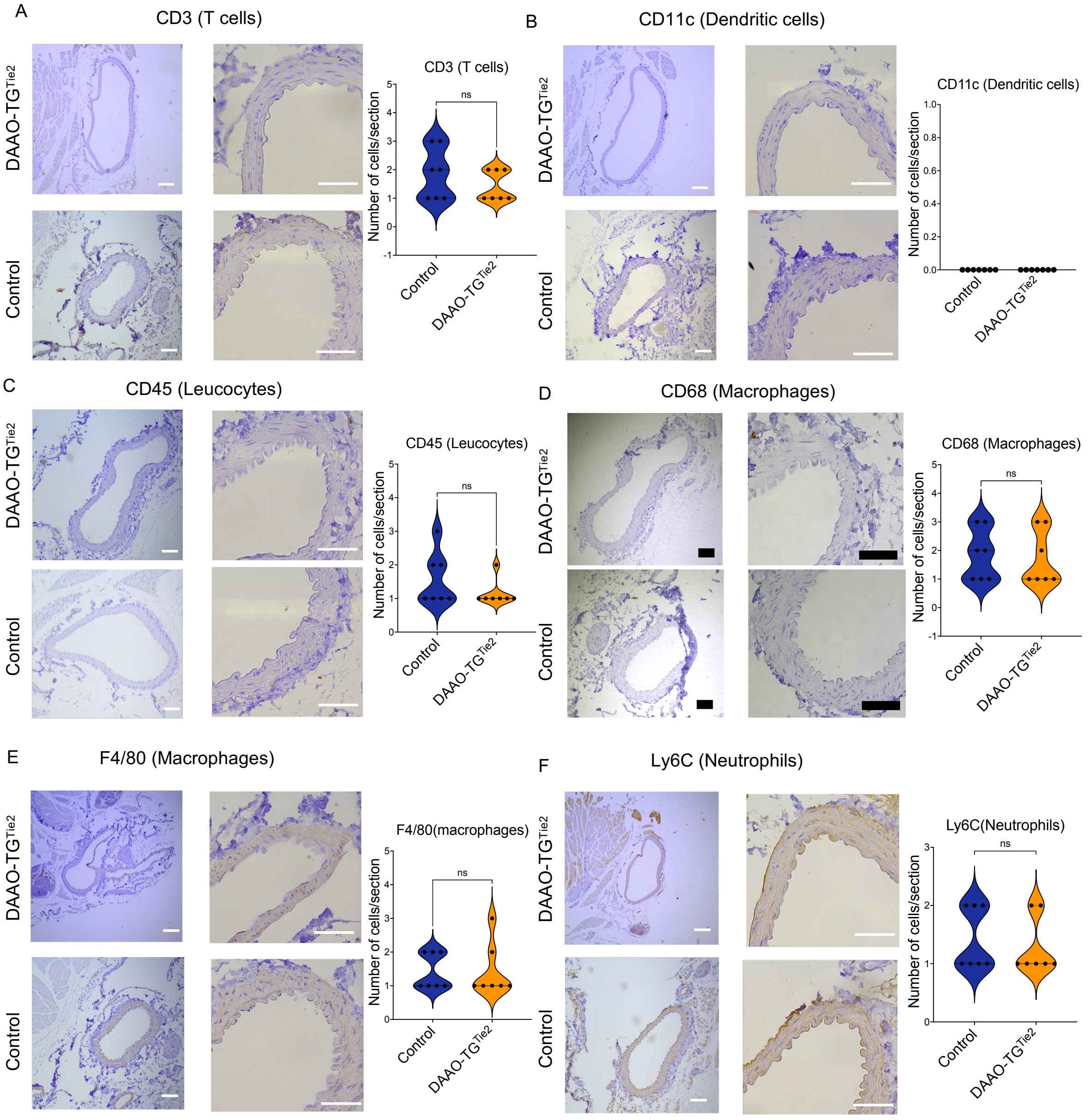

113

Supplemental Figure 5: Immunohistochemical staining of infrarenal abdominal aorta with immune cell markers

This figure shows the results of staining of infra-renal abdominal aortae with a panel of antibodies to characterize the prevalence of immune cells in aortic tissues isolated from D-alanine-treated control or DAAO-TG^{Tie2} mice. After 3 months of D-alanine feeding, animals were euthanized and abdominal aortic tissues were isolated and fixed, and transverse sections were stained with antibodies as noted. For each representative photomicrograph, summary data showing the number of positively-stained cells are shown in violin plots representing the analysis of 7 sections from n=3 mice; observers were blinded to genotype and treatment. **Panel A** shows staining with the T cell marker CD3; **panel B** shows staining with the dendritic cell marker CD11c; **panel C** shows CD45 staining (leukocytes); **panels D and E** show staining with two different macrophage-specific antibodies (CD68 and F4/80, as shown. Panel F shows staining with the neutrophil marker Lys6C. Statistical comparisons are performed with Student's unpaired t test. $p<0.05$ is considered as significant and "ns" indicates not significant. The scale bars show 100 µm. Data shown here represents mean ± SEM of at least 3 independent experiments.

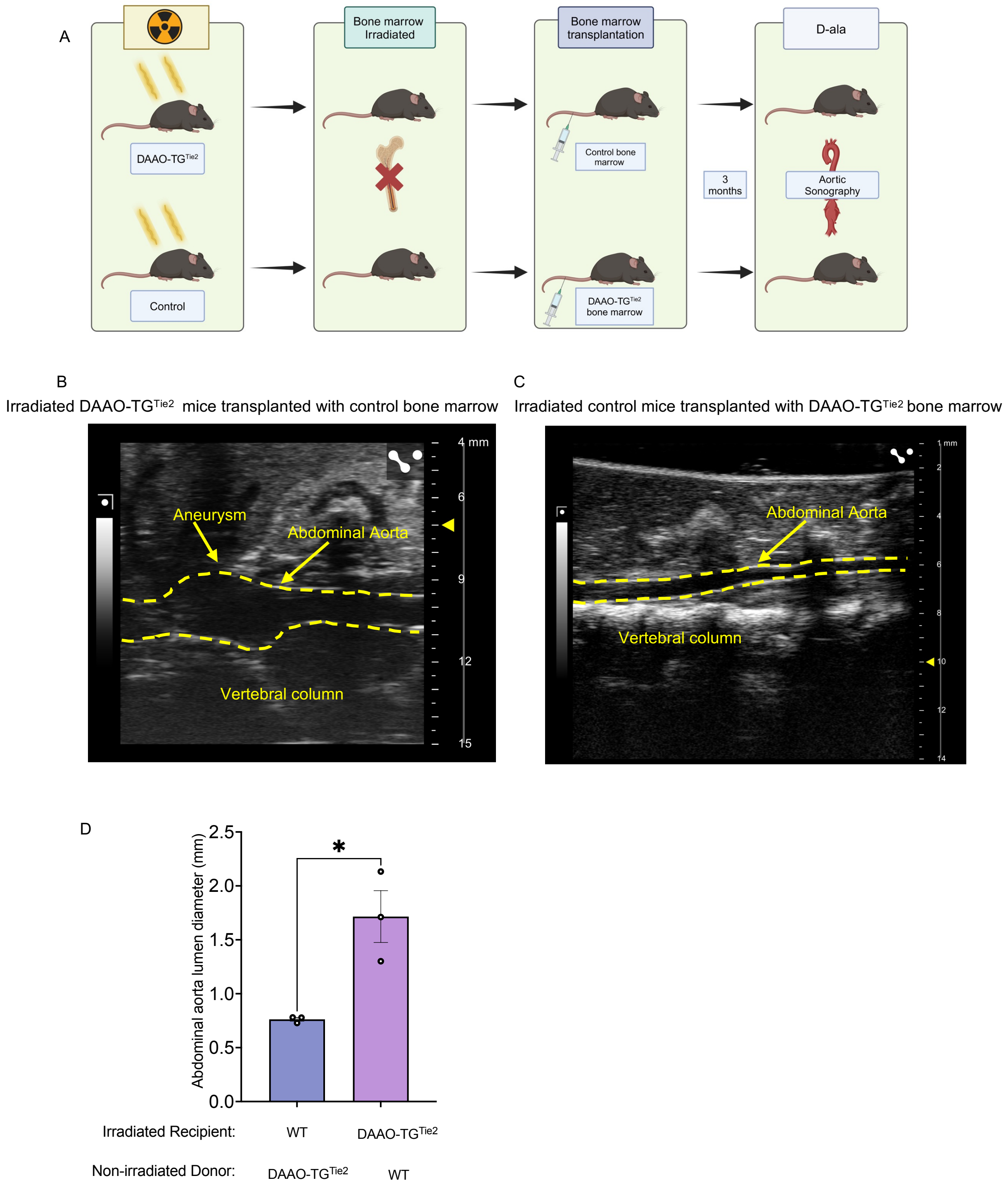
A
Bone marrow Irradiated
Bone marrow transplantation
D-ala
DAAO-TG^{Tie2}
Control
Control bone marrow
DAAO-TG^{Tie2} bone marrow
3 months
Aortic Sonography
B
Irradiated DAAO-TG^{Tie2} mice transplanted with control bone marrow
Aneurysm
Abdominal Aorta
Vertebral column
4 mm
6
9
12
15
C
Irradiated control mice transplanted with DAAO-TG^{Tie2} bone marrow
Abdominal Aorta
Vertebral column
1 mm
2
4
6
8
10
12
14
D
Abdominal aorta lumen diameter (mm)
2.5
2.0
1.5
1.0
0.5
0.0
*
Irradiated Recipient:
WT
DAAO-TG^{Tie2}
Non-irradiated Donor:
DAAO-TG^{Tie2}
WT

Supplemental Figure 6: Results of D-alanine feeding following bone marrow transplants into irradiated control and DAAO-TG^{Tie2} donor mice.

Panel A shows the experimental approach used to determine whether hematopoietic cells convey the vascular pathophenotype. DAAO-TG^{Tie2} or Cre^{+}/TG^{-} control mice were irradiated (11 Gy) to ablate hematopoietic cells; these irradiated mice were then the recipients of bone marrow from non-irradiated control or DAAO-TG^{Tie2} mice, and the transplant recipients were then treated with D-alanine for 3 months. **Panel B** shows a representative aortic sonographic image in D-alanine-fed irradiated DAAO-TG^{Tie2} mice that had been transplanted with bone marrow from non-irradiated control mice, and **panel C** shows results of sonography in irradiated control mice that had been transplanted with bone marrow from non-irradiated DAAO-TG^{Tie2} mice. Abdominal aortic dimensions were measured by blinded observers, and pooled sonographic data are shown in **Panel D**. The * denotes $p<0.05$ (Student's t test) comparing 3 mice in each group.

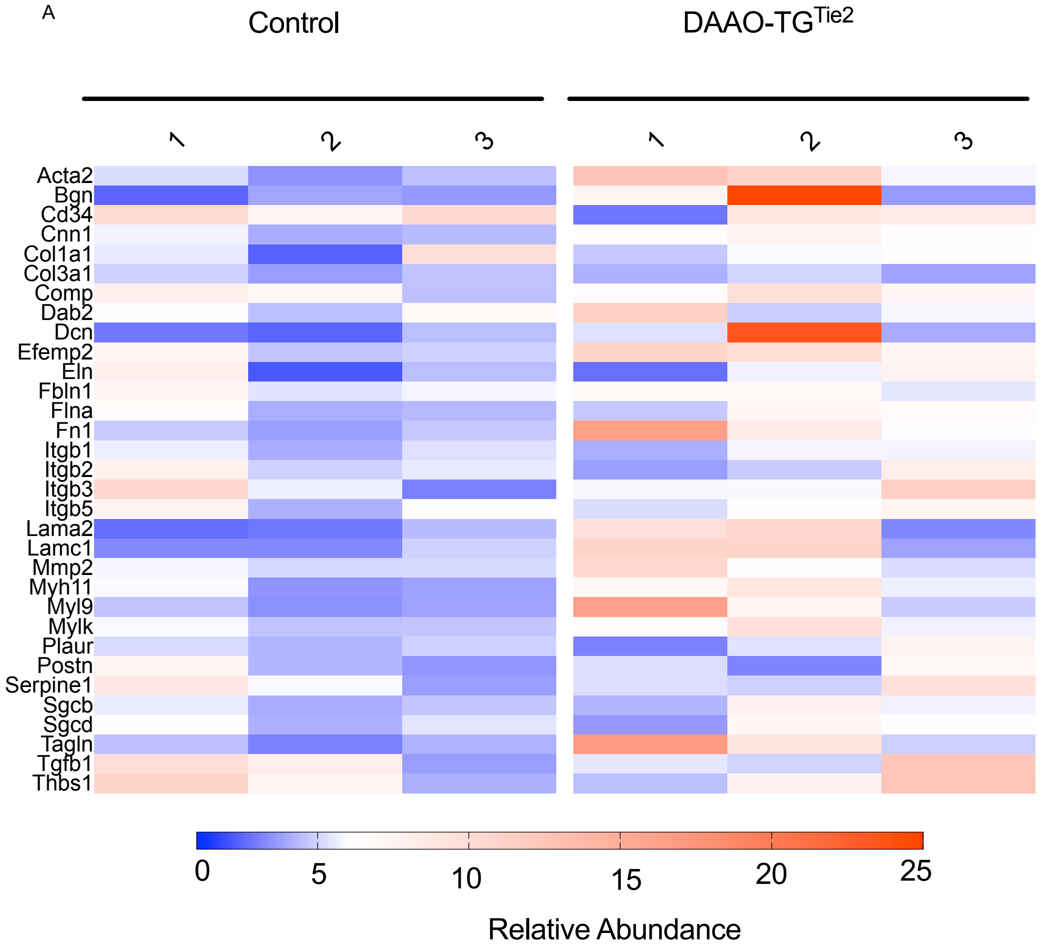

Supplemental Figure 7: Heat map showing relative abundance of EnMT dataset proteins in abdominal aorta in control and DAAO-TG^{Tie2} mice

(A) Abdominal aortae were isolated from D-alanine-fed DAAO-TG^{Tie2} and Cre^{+}/TG^{-} control mice, and comparative proteomics analyses were performed to determine the relative protein abundance for a panel of EnMT pathway proteins following D-alanine feeding. Individual proteins are identified for each row, and the columns represent the results for individual mice (n=3 each treatment). Blue color represents decreases in protein abundance and the red color indicates increases in abundance. Data shown here represents results of at least 3 independent experiments.

A

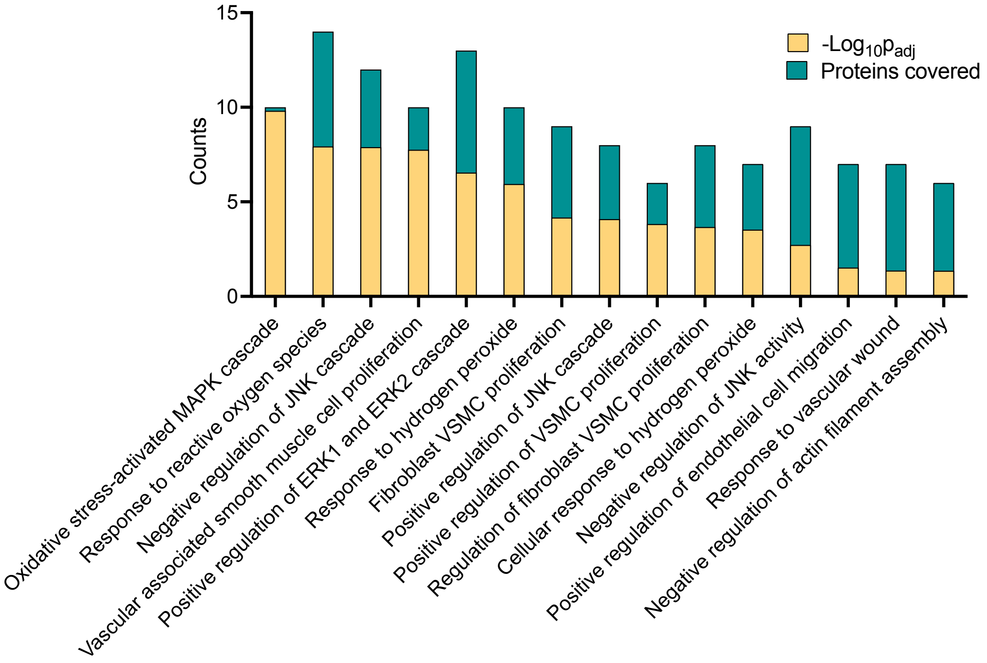
-Log10padj
Proteins covered
Counts
15
10
5
0
Oxidative stress-activated MAPK cascade
Response to reactive oxygen species
Negative regulation of JNK cascade
Vascular associated smooth muscle cell proliferation
Positive regulation of ERK1 and ERK2 cascade
Response to hydrogen peroxide
Fibroblast VSMC proliferation
Positive regulation of JNK cascade
Positive regulation of VSMC proliferation
Regulation of fibroblast VSMC proliferation
Cellular response to hydrogen peroxide
Negative regulation of JNK activity
Positive regulation of endothelial cell migration
Response to vascular wound
Negative regulation of actin filament assembly

B

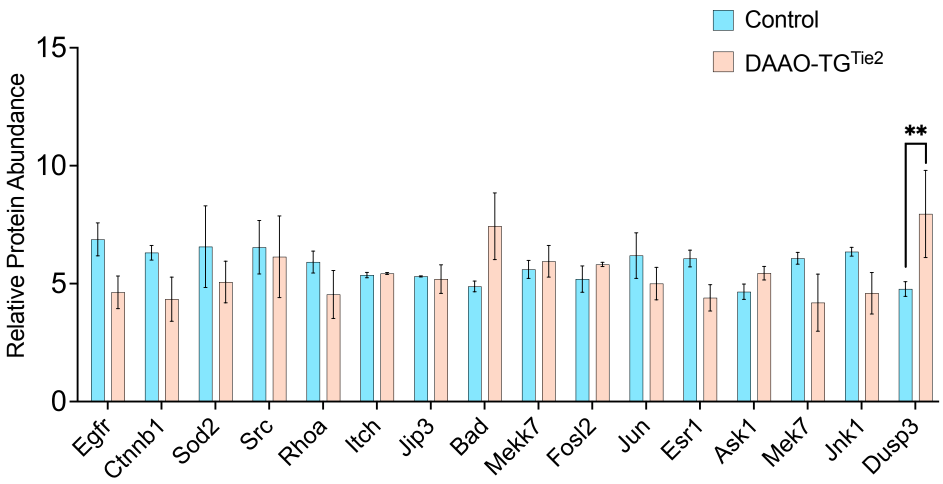
Control
DAAO-TGTie2
Relative Protein Abundance
15
10
5
0
**
Egfr
Ctnnb1
Sod2
Src
Rhoa
Itch
Jip3
Bad
Mekk7
Fosl2
Jun
Esr1
Ask1
Mek7
Jnk1
Dusp3

153

154

Supplemental Figure 8: Enriched Gene Ontology Biological Processes analyzed from the JNK1 network dataset

Panel A shows the most significant Gene Ontology (GO): Biological Processes values, plotted as stacked columns with $-\log_{10}p_{adj}$ values shown in yellow and the number of proteins covered within each analysis shown in blue; the names of individual Biological Processes are listed along the X axis. **Panel B** shows the relative abundance of the most relevant proteins in Gene Ontology: Biological Processes analysis within the JNK1 network dataset determined after D-alanine treatment for both DAAO-TG^{Tie2} transgenic mice and their control littermates. The ** denotes $p<0.01$ (unpaired Student's t test, n=3 mice in each group).

References

1. Yadav S, et al. Sensory ataxia and cardiac hypertrophy caused by neurovascular oxidative stress in chemogenetic transgenic mouse lines. Nat Commun. 2023;14(1):3094.
2. Payne S, De Val S, Neal A. Endothelial-Specific Cre Mouse Models. Arterioscler Thromb Vasc Biol. 2018;38(11):2550–2561.
