# Supplementary material for: Differential aortic aneurysm formation provoked by chemogenetic oxidative stress
Source: J Clin Invest. 2025 Mar 18;135(9):e188743. doi: 10.1172/JCI188743 (PMC12043099; doi:10.1172/JCI188743)
Supplement: Unedited blot and gel images [file jci-135-188743-s166.pdf]

FULL BLOTS

FIGURE 6A AND SUPPLEMNTAL FIG 3B

ASK1-Phospho: FULL BLOT (FIG 6 A)

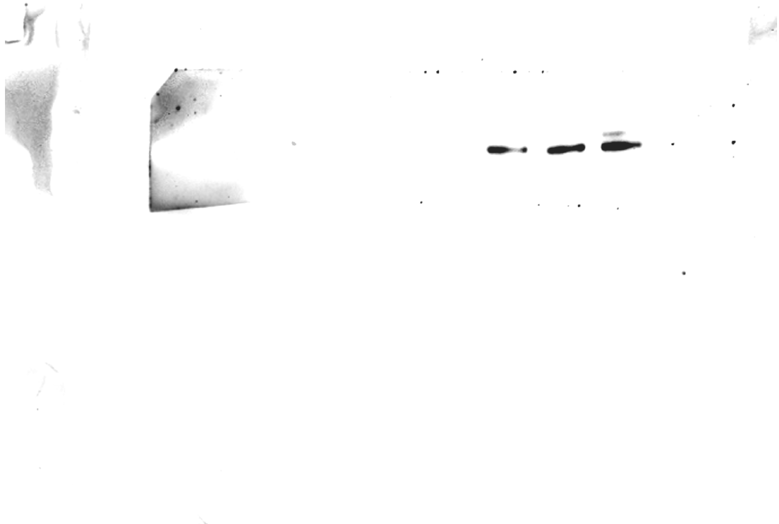

ASK1 phospho Abdominal

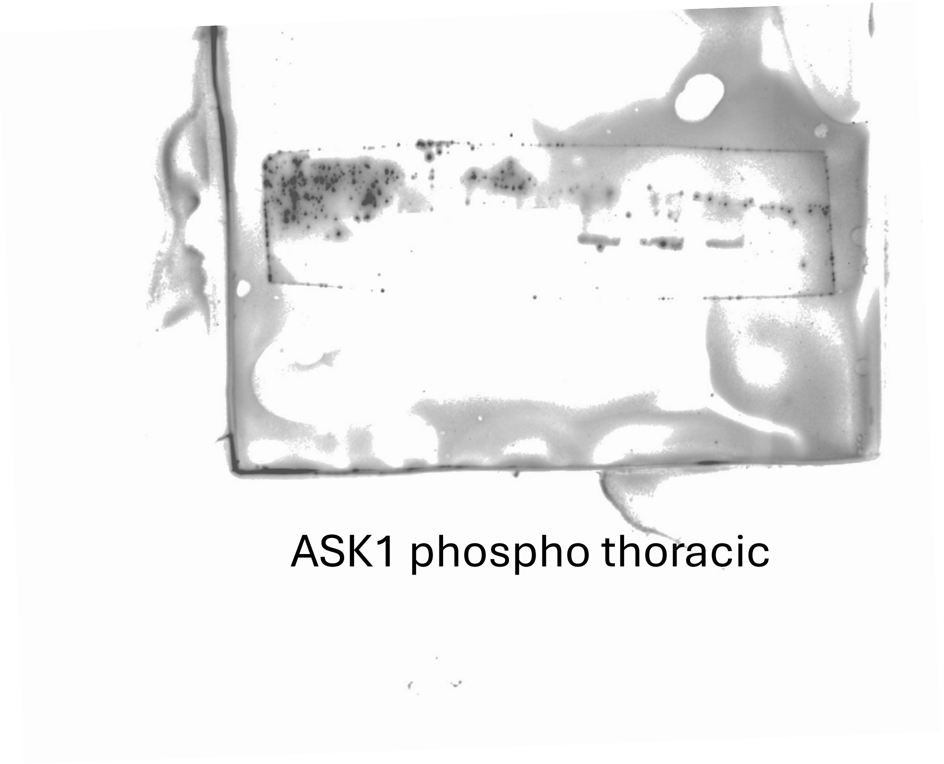

ASK1 phospho thoracic

ASK1 Total: FULL BLOT (FIG 6 A)

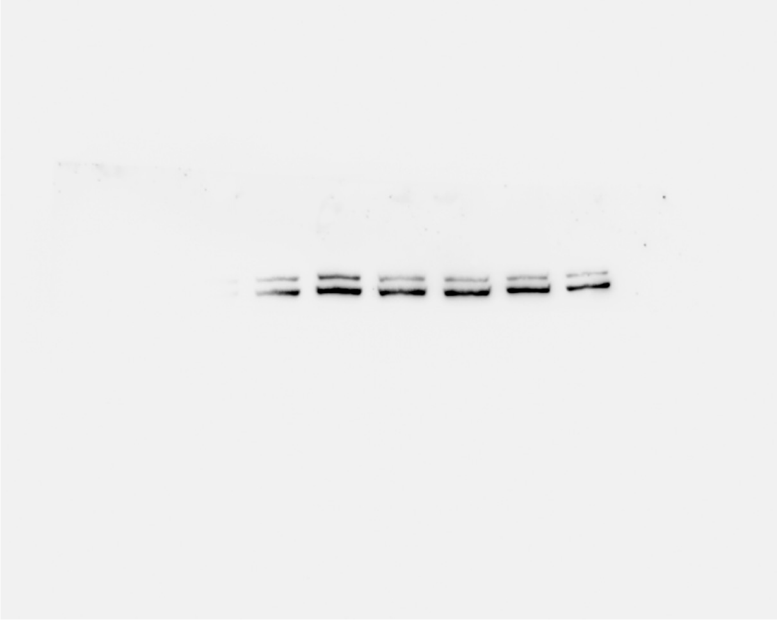

ASK1 total abdominal

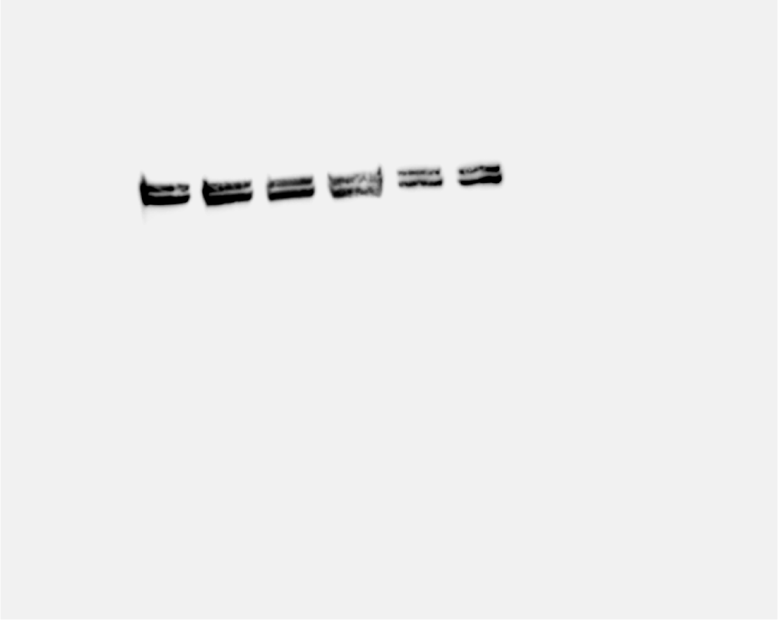

ASK1 total thoracic

DUSP3: FULL BLOT (FIG 6 A)

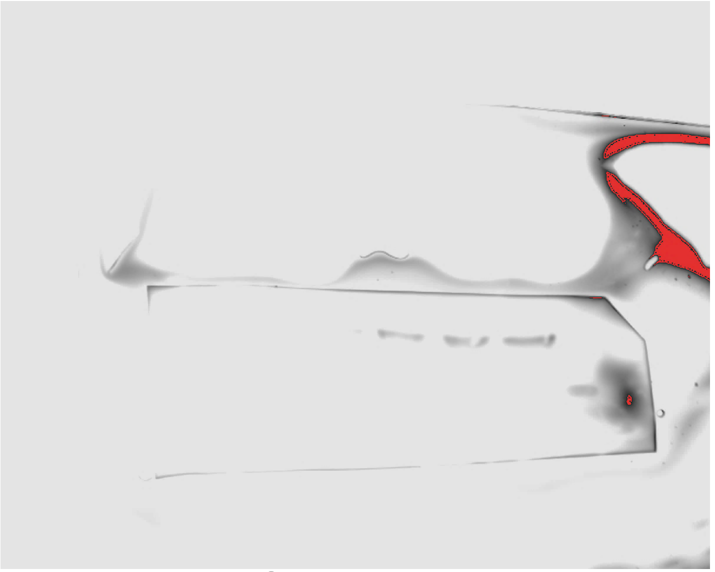

DUSP3 abdominal

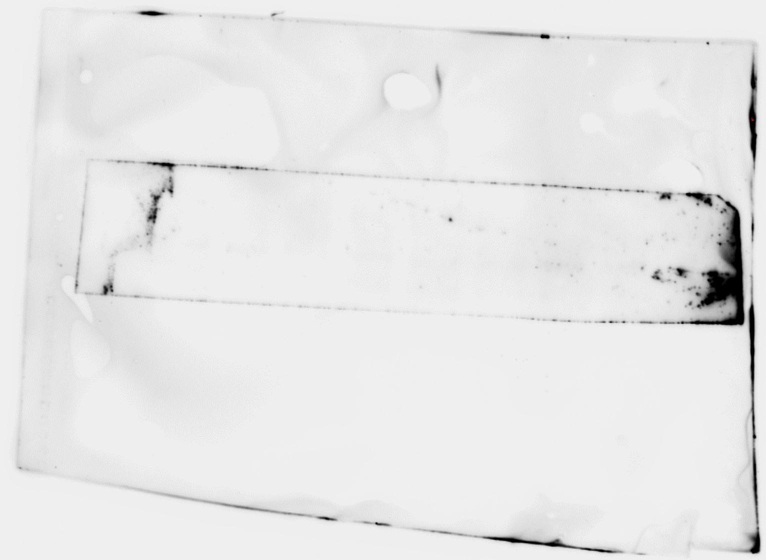

DUSP3 thoracic

Vinculin: FULL BLOT (FIG 6 A)

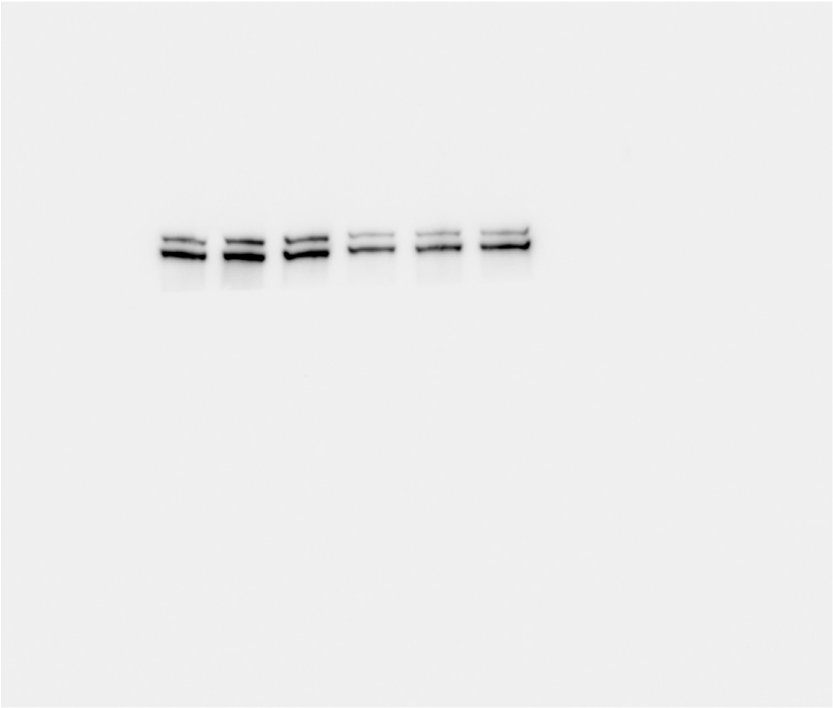

Vinculin abdominal

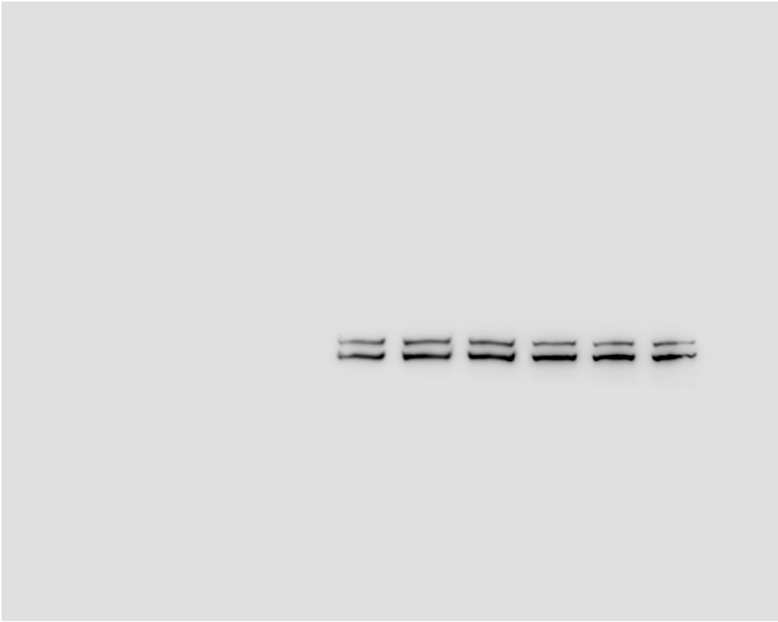

Vinculin Thoracic

MEK7-Phospho: FULL BLOT (FIG 6 A)

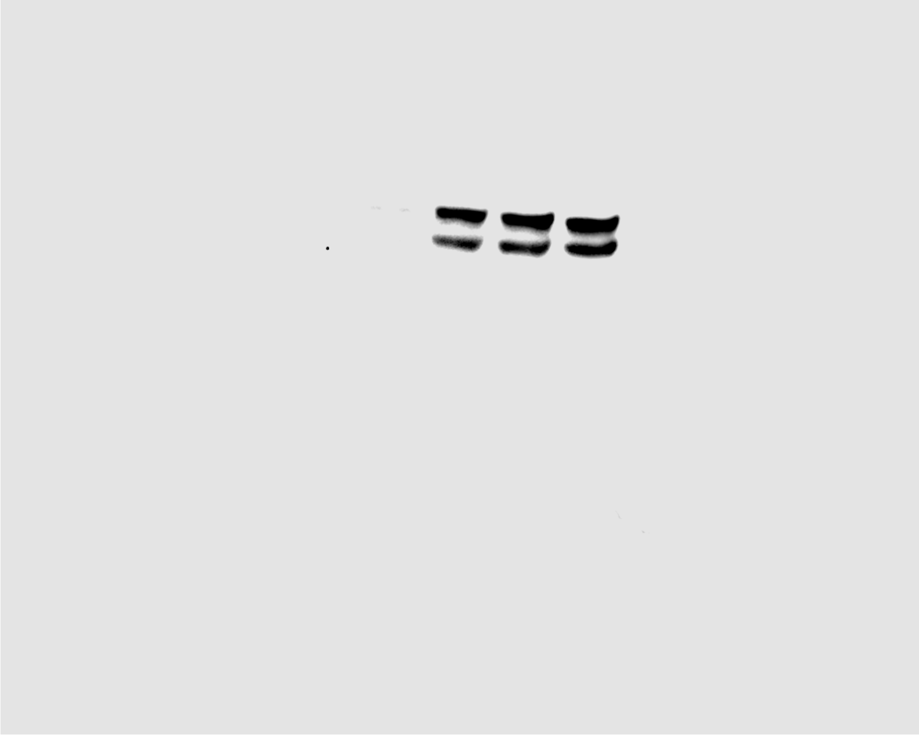

Phospho-MEK7 abdominal

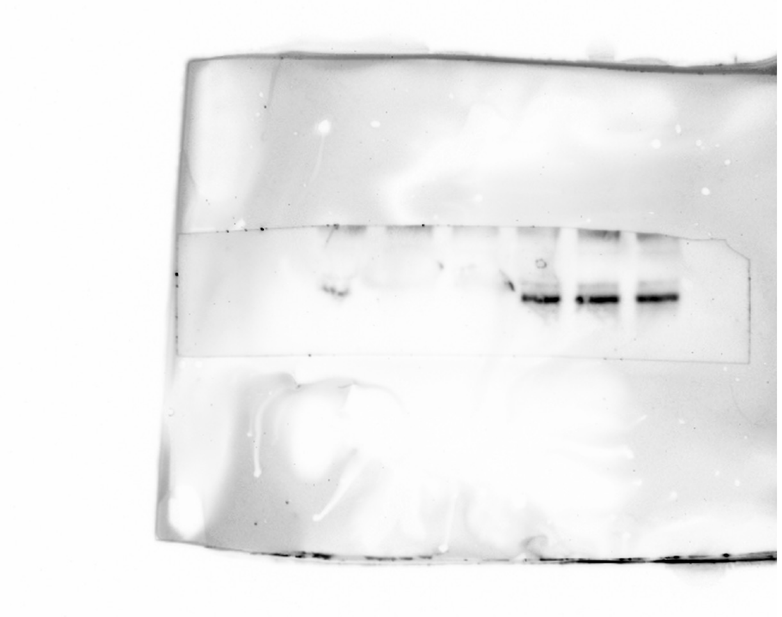

Phospho-MEK7 thoracic

Total MEK7: FULL BLOT (FIG 6 A)

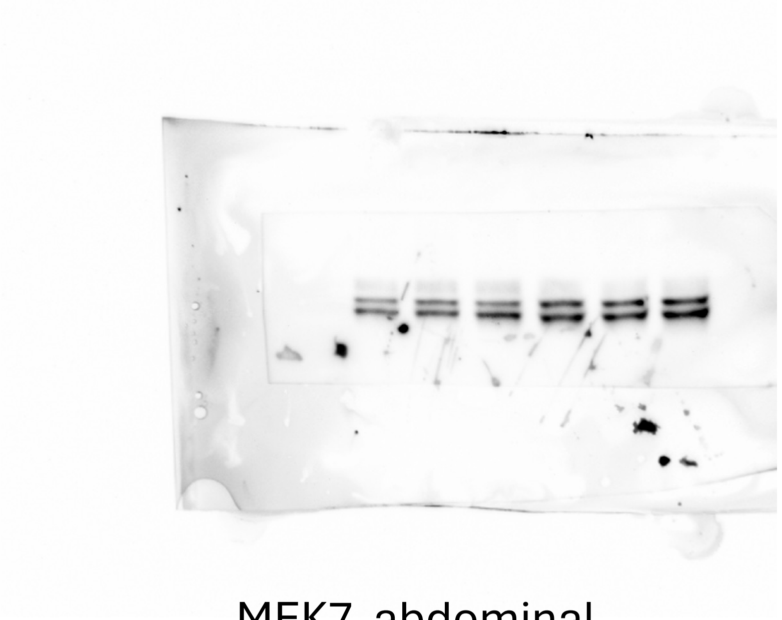

MEK7 abdominal

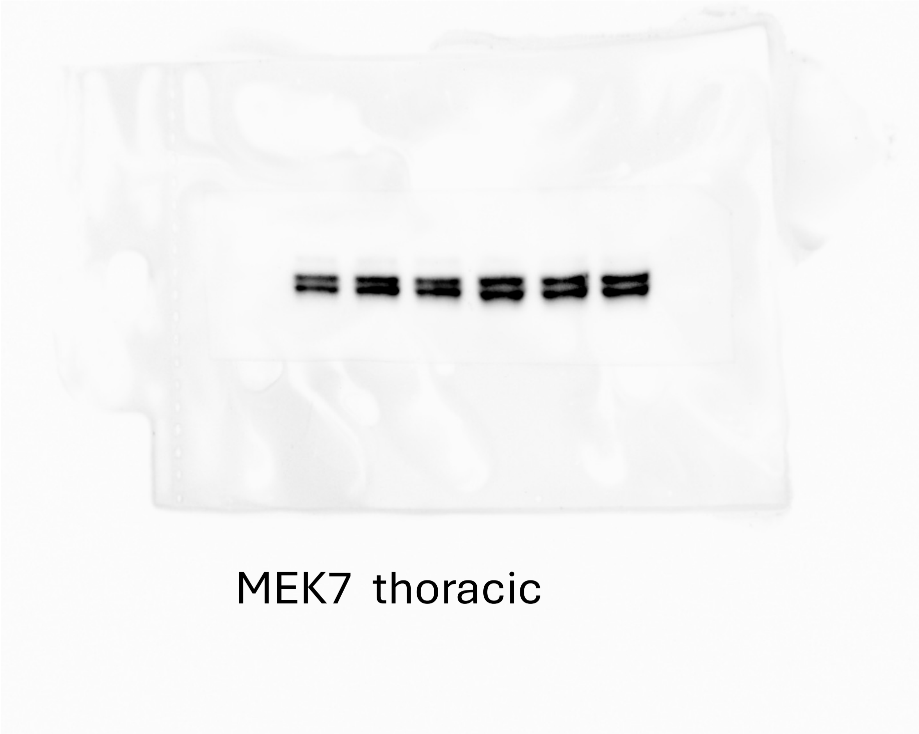

MEK7 thoracic

Phospho-JNK1: FULL BLOT (FIG 6 A)

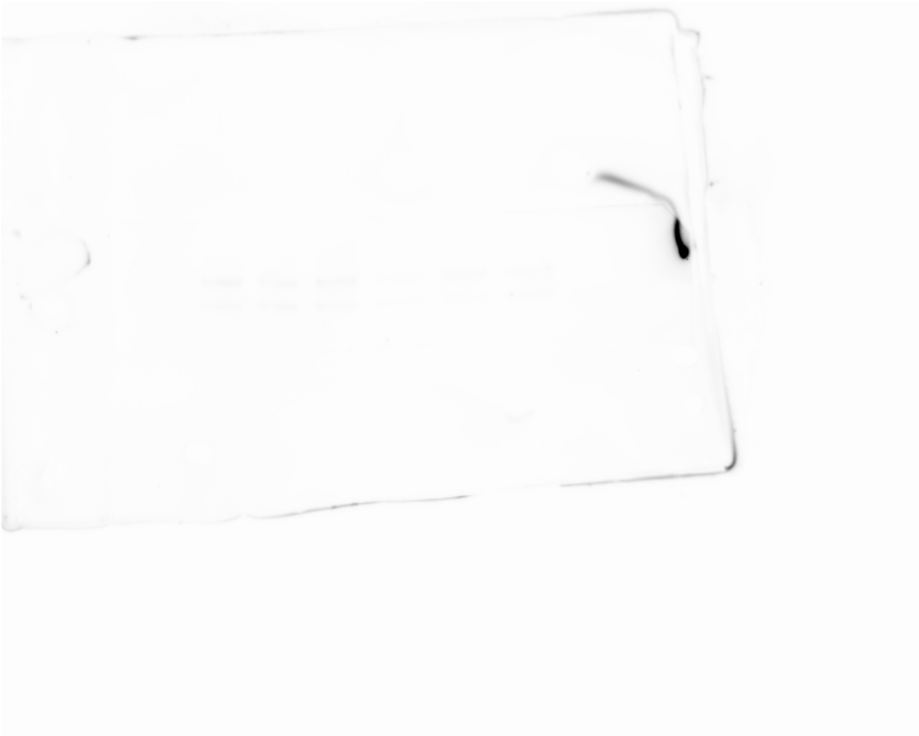

Phospho-JNK1 abdominal

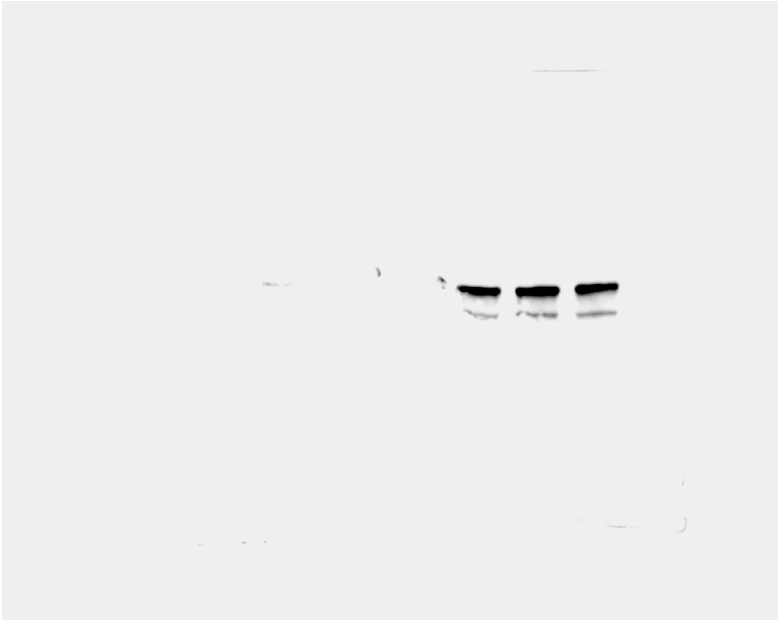

Phospho-JNK1 thoracic

JNK1 Total: FULL BLOT (FIG 6 A)

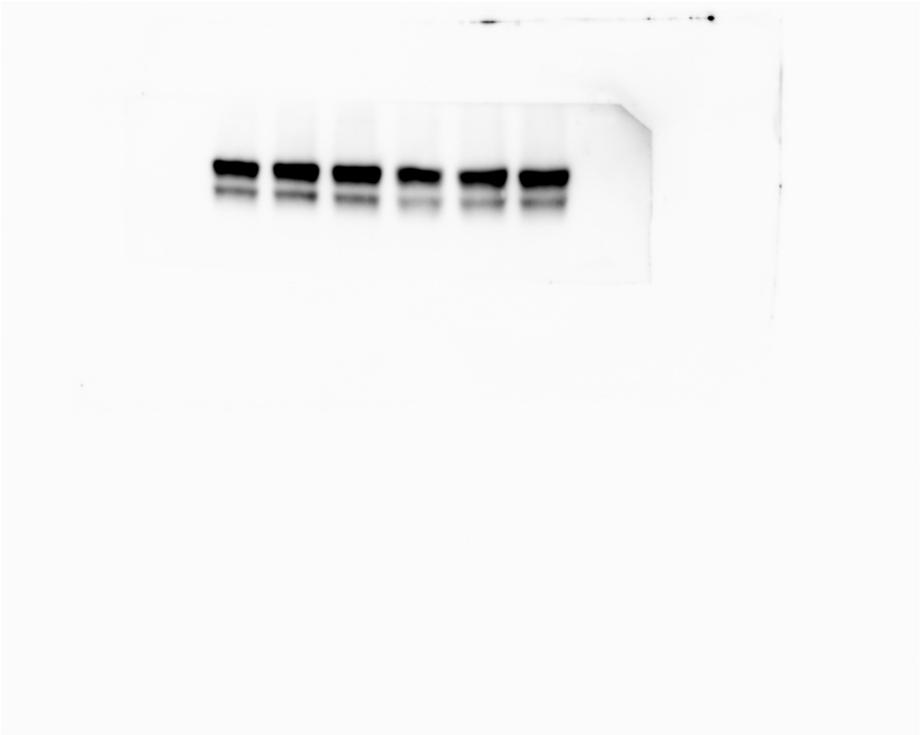

Total JNK1 abdominal

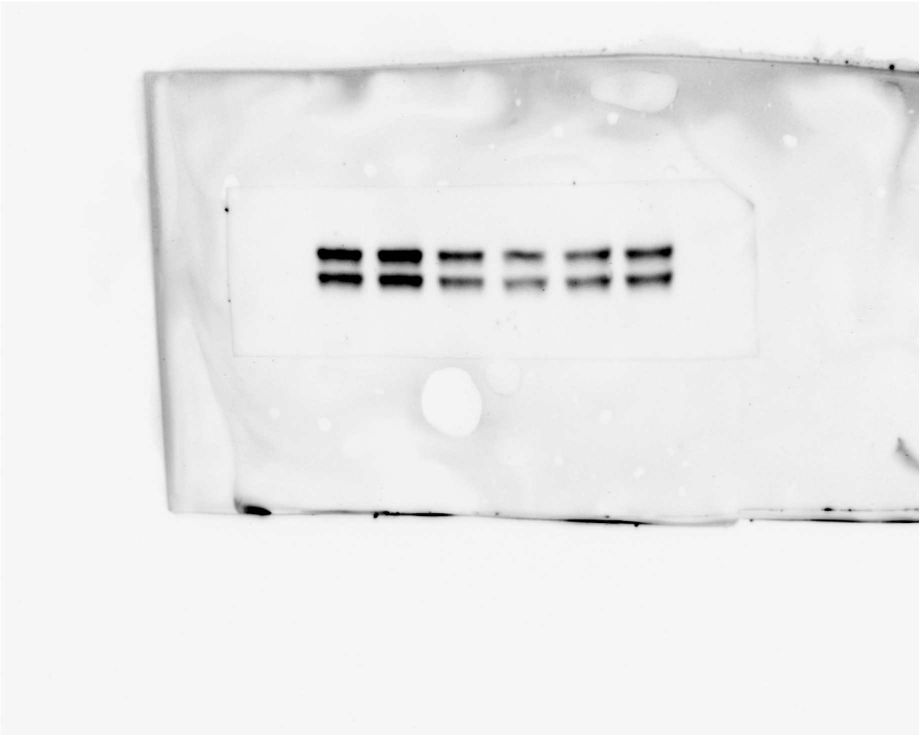

Total JNK1 thoracic

GFP and corresponding Vinculin: FULL BLOT (SUP FIG 3B)

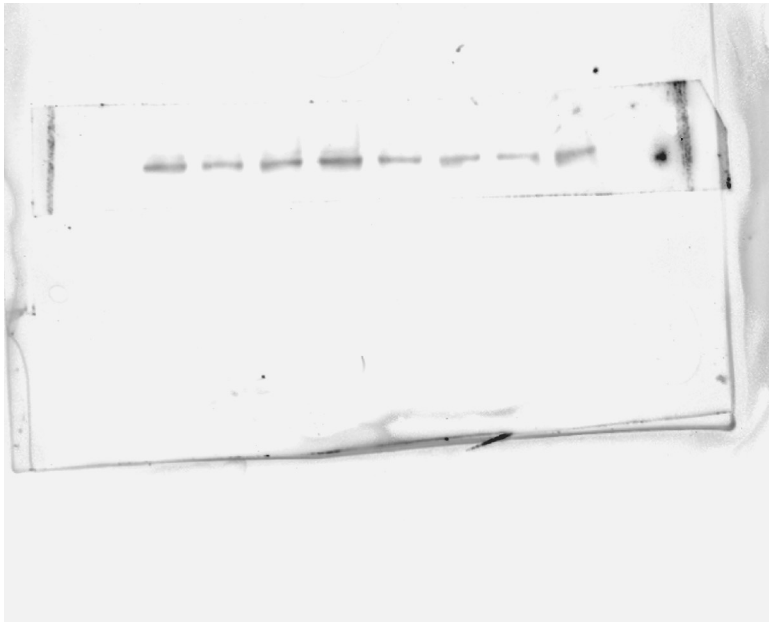

GFP

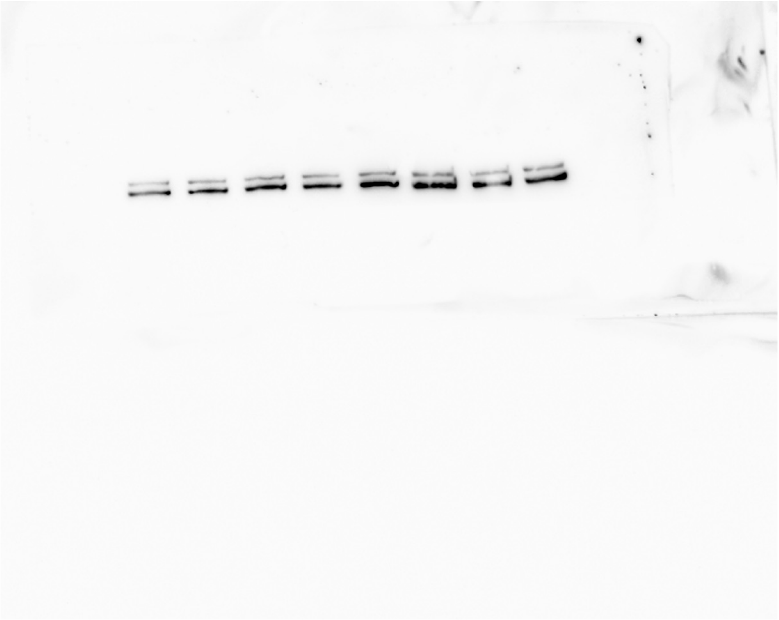

Vinculin
